# Expression divergence measured by transcriptome sequencing of four yeast species

**DOI:** 10.1186/1471-2164-12-635

**Published:** 2011-12-29

**Authors:** Michele A Busby, Jesse M Gray, Allen M Costa, Chip Stewart, Michael P Stromberg, Derek Barnett, Jeffrey H Chuang, Michael Springer, Gabor T Marth

**Affiliations:** 1 Department of Biology, Boston College, 140 Commonwealth Avenue, Chestnut Hill, MA 02467-3961, USA; 2 Department of Neurobiology, Harvard Medical School, 220 Longwood Avenue, Boston, Massachusetts 02115, USA; 3 Department of Systems Biology, Harvard Medical School, 200 Longwood Avenue, Boston, MA 02115, USA; 4 Department of Genetics, Harvard Medical School 77 Avenue Louis Pasteur, NRB 0356, Boston, MA 02115, USA; 5 Broad Institute of Harvard and MIT, 7 Cambridge Center, Cambridge, MA 02142

**Keywords:** RNA-Seq, Comparative transcriptomics, *S. cerevisiae*, *S. paradoxus*, *S. mikatae*, *S. bayanus*

## Abstract

**Background:**

The evolution of gene expression is a challenging problem in evolutionary biology, for which accurate, well-calibrated measurements and methods are crucial.

**Results:**

We quantified gene expression with whole-transcriptome sequencing in four diploid, prototrophic strains of *Saccharomyces *species grown under the same condition to investigate the evolution of gene expression. We found that variation in expression is gene-dependent with large variations in each gene's expression between replicates of the same species. This confounds the identification of genes differentially expressed across species. To address this, we developed a statistical approach to establish significance bounds for inter-species differential expression in RNA-Seq data based on the variance measured across biological replicates. This metric estimates the combined effects of technical and environmental variance, as well as Poisson sampling noise by isolating each component. Despite a paucity of large expression changes, we found a strong correlation between the variance of gene expression change and species divergence (R^2 ^= 0.90).

**Conclusion:**

We provide an improved methodology for measuring gene expression changes in evolutionary diverged species using RNA Seq, where experimental artifacts can mimic evolutionary effects.

GEO Accession Number: GSE32679

## Background

Previous studies have found that gene expression diverges as the distance between species increases, and that this divergence is linearly related to the time since divergence (Reviewed in [1]). This expression evolution has been reported to be slow enough that orthologous genes still have highly correlated expression, even in species that diverged up to 400 million years ago [2,3]. Several studies have reported that genes with specific attributes change expression more quickly [4-7], though it is not known whether the expression of such subsets of genes also diverges linearly with time.

RNA-Seq offers a methodological improvement over microarrays for measuring expression divergence because it does not suffer from the probe-based biases that confound cross-species microarray measurements [8-11]. Technical replication studies, in which expression values are assayed more than once from the same sample, have shown that RNA-Seq quantifies relative gene expression accurately [12,13].

However, measuring gene expression by RNA-Seq is complicated by alternate sources of variance [14]. The gross inter-species measurement of gene expression by RNA-Seq contains four components: (1) true inter-species gene expression difference; (2) expression differences caused by environmental variance; (3) variance from technical measurement imprecision; and (4) Poisson sampling noise. Because of these alternate sources of variance, it is possible for a gene without true inter-species gene expression changes to be measured with different values in different species.

Therefore, we first developed methods to quantify biologically relevant expression differences between species that are greater than the variance within each species by collecting expression data from biological replicates of the well-characterized model organism *S. cerevisiae *and the three related yeasts: *S. paradoxus, S. mikatae*, and *S. bayanus *[15]. In all cases we used diploid, prototrophic strains of the yeast. This approach ensures that we are measuring the expression change that can be attributed to evolution rather than artificial expression changes that occur due to the disruption of the auxotrophic pathways.

We applied our methods to identify differentially expressed (DE) genes and examine the rate and properties of expression evolution in yeast.

## Results

We grew two independent cultures of each the following prototophic, diploid strains: *S. cerevisiae *(FY4 (MAT a) and FY5 (MAT alpha) [16] which we mated to get a diploid, *S. paradoxus *(Y-17217), *S. mikatae *(IFO1815), and *S. bayanus *(MCYC623) according to the protocols described in Methods. RNA was isolated and sequenced using a strand-specific protocol. A total of over 292 million 35 base pair reads were generated between two runs of an AB SOLID sequencer [17]. A third sample of *S. cerevisiae *was also grown and sequenced as a pair of technical replicates.

Over 156 million single end AB SOLiD reads aligned to their respective genomes (54%) using the MOSAIK alignment program (http://bioinformatics.bc.edu/marthlab/Mosaik) as described in Methods and Additional File 1, Supplemental Methods 1. The reference genome and annotations for *S. cerevisiae *were downloaded from the *Saccharomyces *Genome Database (http://downloads.yeastgenome.org/). Genome, annotations, and orthology mappings for the other species were from Kellis et al. [15] (Additional File 1, Supplemental Methods 1).

Of these aligned reads, ^~^113 million (69%) aligned uniquely. The dominant source of unaligned reads was sequencing errors (Additional File 1, Supplemental Methods 2). We examined the effect of reference quality and found that alignment rates increased as reference genomes were sequenced to higher depths (Additional File 1, Table S1).

Not all genes can be measured accurately using RNA-Seq, particularly in cross-species comparisons where the length of genes and the percentage of genes that can be uniquely aligned can vary between species. When comparing the expression in 1:1 orthologs across species, we selected genes where we were confident that accurate measurements could be found (Methods). Genes that were annotated with measureable orthologs in all four species were designated as "core" genes.

### Environmental variance accounts for up to 60% of the expression variance observed in inter-species comparisons

**Figure 1:**
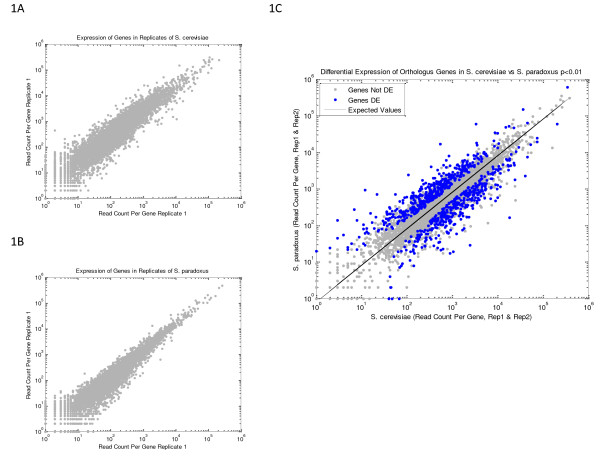
**Gene expression measurements in replicates of *S. cerevisiae *(1a) and S. *paradoxus *(1b), and the comparison between these two species (1c)**. Plotted values are the number of reads uniquely aligning to the gene. Differentially expressed genes are indentified (blue) as p < 0.01 based on our Χ^2 ^metric.

We compared the differences in expression observed in different species to the differences in expression observed in biological replicates of the same species to determine what portion of the variance is due to genomic variation, versus environmental and measurement variance. Expression is highly correlated between orthologous genes in *S. cerevisiae *vs. *S. paradoxus *(Pearson correlation = 0.85). Correlation between biological replicates of *S. cerevisiae *(0.91) and *S. paradoxus *(0.96) suggest that up to 60% of the observed variance between samples of two separate species can be attributed to environmental response and measurement imprecision (Figure 1a, b).

**Figure 2:**
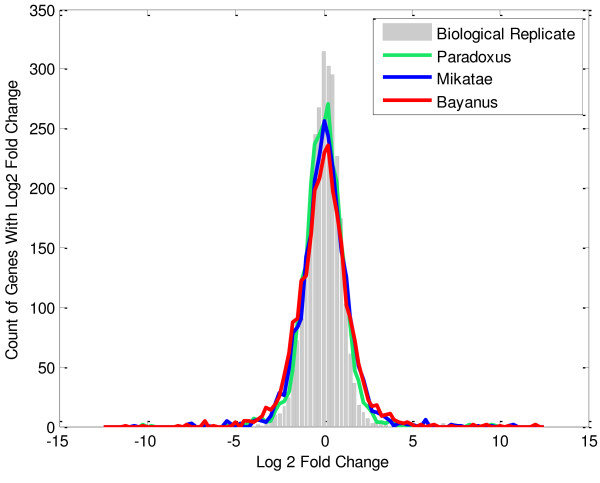
**The distribution of log2 fold changes when the expression levels from each comparison sample are compared to *S. cerevisiae *replicate 1, using the core genes**. Biological replicates are *S. cerevisiae *replicate 2 versus *S. cerevisiae *replicate 1. Interspecies comparisons are replicate 1 of the comparison species versus *S. cerevisiae *replicate 1.

The distribution of the log2 fold changes observed between species also shows a high level of overlap with the distribution of fold changes between biological replicates, with the distribution widening as the comparison species become more diverged (Figure 2). For example, in the biological replicates of *S. cerevisiae*, 95% of the genes show a log2 fold-change in the range of [-1.9, 1.9]. Even in the most distant interspecies comparison (*S. bayanus *vs. *S. cerevisiae*), 84% of the fold changes were still within this range.

### Source of variance in expression measurements

We then examined the sources of variation in expression measurements by comparing technical and biological replicates. Technical replicates were prepared from RNA isolated from a single culture of *S. cerevisiae *that was divided into two samples that were separately processed through the entire library preparation and sequencing protocol. Biological replicates were prepared from two separate cultures of *S. cerevisiae*. Technical replicates contain variance from measurement imprecision and Poisson counting noise but no environmental variance. Biological replicates contain counting noise, measurement imprecision, and environmental variance but not true inter-species gene expression differences.

We compared the reproducibility of measurements of the same gene in technical versus biological replicates of S. cerevisiae using fold change as our metric (Additional File 1, Table S2 and Figure S1A and S1B). Within the technical replicates, we found that coding genes showed better reproducibility than non-coding genes, which were comprised mainly of snRNAs, snoRNAs, and tRNAs (Additional File 1, Figure S1C). Non-coding genes were therefore excluded from further analyses. To isolate and quantify the effects of technical and biological variance, we excluded genes which were measured with fewer than ten reads. At low counts, Poisson variance is high relative to the total read count, and Poisson fluctuations can cause large fold changes (Additional File 1, Figure S2). Poisson error is lower in genes measured with at least ten reads, which is true for > 95% of our core genes, in all samples used for DE calls. When we considered genes with at least ten reads in each condition, ninety-five percent of gene expression measurements reproduced within a 1.8x fold-change between the two technical replicates. In biological replicates, 95% of genes reproduced within 3.6x fold change, but a small number of genes showed large expression differences with fold changes as high as 25x. While technical variance is low, it acts subsequent to biological variance, and can therefore amplify or dampen the fold changes that occur to due to environmental variance. We note that fold changes measured with RNA-Seq are expected be higher than comparable measurements taken with microarrays due to the limited dynamic range inherent in microarrays [18]. We show estimated conversion factors in Additional File 1, Figure S3.

To demonstrate that the variance that we observed in our biological replicates occurred due to responses to normal laboratory conditions, rather than a poorly controlled environment or unusually imprecise RNA-Seq measurements, we compared our results to the correlation of *S. cerevisiae *replicates measured by RNA-Seq using Illumina technology in Nagalakshmi et al. [13]. The R^2 ^value of log2 expression values of our biological replicates ranged from 0.867 for the replicates of *S. cerevisiae *to 0.900 for *S. paradoxus*. The corresponding values reported in Nagalakshmi et al. are nearly identical (R^2 ^= 0.869 and R^2 ^= 0.904). We then compared the fold changes that occurred between repeated measurements of the same gene in biological replicates and found that, while differences exist between replicate runs, our results were comparable to the results from Nagalakshmi (Additional File 1, Figure S4).

Because samples were grown on different days, we examined whether batch effects could introduce an additional source of variability in gene expression measurements. We identified a set of 162 genes that appeared to have higher expression in the second replicate of S. paradoxus versus the first. We found that the orthologs of these genes were also disproportionately up-regulated in the second replicate of S. cerevisiae (90%), S. mikatae (62%), and S. bayanus (96%) (Additional File 1, Figure S5). These genes showed enrichment for involvement in RNA metabolic processes (p < 0.01). Therefore, in subsequent analyses comparisons were only made between samples which were grown within the same batch.

This reveals the difficulty of calling DE genes in comparisons of samples separated by evolutionary distances: the signal (true differential expression due to genetic difference) to noise (environmental variance, technical variance, and Poisson noise) ratio is very low.

### Fold change measurements obtained from RNA-Seq are confirmed with qPCR

To demonstrate the accuracy of our gene expression measurements, we compared FC measurements obtained with RNA-Seq to those obtained using qPCR (Additional File 1, Supplemental Methods 3). In order to adequately quantify the effects of environmental variance, the qPCR was run on three additional biological replicates that were not those used for RNA-Seq (Additional File 1, Figure S6 and Table S3). Additional File 1, Figure S6A shows the results obtained in the comparison between *S. cerevisiae *and *S. mikatae*, which is representative of the results found in all six of the cross-species comparisons. Due to the response of genes to subtle environmental differences and measurement imprecision in both technologies, the 2σ (95%) confidence intervals around the FC measurements in each test span multiple log2 fold-changes for both RNA-Seq and qPCR. In this validation, 22 out of the 27 measurements (81%) of the qPCR measurements are within the boundaries of the fold change intervals for both RNA-Seq fold change measurements. While this result is somewhat below expectations, we note that measurements are not independent and single outlier measurements can cause validation failure for multiple tests.

### Identifying differentially expressed genes using a Χ^2 ^test

To identify genes that are differentially expressed to a statistically significant degree, we developed a Χ^2 ^test that models the combination of environmental, technical, and Poisson variance as Poisson fluctuation with over-dispersion (Methods).

To test the sensitivity of our method, we used simulations of RNA-Seq data based on distributions measured in the actual data (Additional File 1, Supplemental Methods 4). We tested our method against a paired t-test and DESeq [19] run with the default settings. While there are many differences between the methods, all test whether the difference in the measured expression between the two species is greater than the variance. The methods have fundamental differences in the value that they use as the variance. A paired t-test uses the measured variance, and thus provides an unbiased result and p-values that closely match the false positive rate of the dataset. However, because variance is poorly measured with only two replicates, the power of the t-test is relatively low (Additional File 1, Figure S7A). The other methods integrate additional information into the estimate of variance, which improve their power but can bias the results. In our method, we assumed that the variance for a given gene will be conserved across all four species. Our main variance measurement is therefore the mean measured variance of the gene across the four species, which is calculated as described in Methods. Additionally, we used the knowledge that the variance of some genes will be measured at values lower than the true variance due to random fluctuations. We corrected these measurements by replacing ones where the measured variance is below the value of the variance observed in technical replicates with that value. We did this under the assumption that the true variance cannot be lower than the resolution of the technology (Methods). DESeq estimates variance by sharing information between different genes that are expressed at a similar expression level. In simulations, we found that when differences in expression were modeled as random changes of a consistent effect size, as defined by Cohen's D, the power of our method was comparable power to DESeq (Additional File 1, Figure S7A). We found that the false positives called by DESeq were more enriched for high variance genes than those called by our method (Additional File 1, Figure S7B). Both DESeq and our method showed small deviations between the p-value calculated and the actual false positive rate (Additional File 1, Figure S7C). In the actual data, we found that DESeq identified a greater number of genes as differentially expressed than our method (Additional File 1, Table S4). In particular, our method was less likely to identify genes with high measured variance as differentially expressed (Supplementary Figure 7D). We believe that our conservative approach is justified by the fact that genes with high variance tended to also show deviations from qPCR measurements in validation experiments (Additional File 1, Figure S6). Additionally, we used the mean expression and variance of biological replicates that was reported by DESeq to test whether the variances used in the differential expression calls would produce confidence interval boundaries consistent with the qPCR measurements. We found that qPCR measurements fell within the boundaries of the 95% DESeq confidence intervals in 83% of the 162 tests. The comparable metric for our variance measurement was 91% (Additional File 1, Supplemental Methods 3). We also tested our metric against a Poisson exact test [20] and a Fisher exact test, but were not able to obtain a false positive rate lower than 10% with either test, even with a p-value cutoff of 10^-10^.

A Χ^2 ^test that uses the expected value as the denominator (see Methods) would call 4% of the measured genes DE at p < 0.01 in the technical replicates of *S. cerevisiae*, 58% in the biological replicates of *S. cerevisiae*, and 81% in the comparison between *S. cerevisiae *and *S. bayanus*. These results indicate that even in technical replicates the variance between samples is substantially over-dispersed relative to Poisson.

Using our Χ^2 ^metric, we found that the percentage of measured genes that are called DE in cross-species comparisons ranged from 19-25%, at a p-value < 0.01 (Table 1, Additional File 2). At this p-value cutoff, the false discovery rate is low for all comparisons (3.9-5.1%). Figure 1C and Additional File 1, Figure S8 show that differentially expressed genes are far from the null model, even at the highest level of expression.

**Table 1:** Genes Differentially Expressed By Interspecies Comparison

	Intergenic Substitution Rate	All Genes Measured	DE	%DE	Core Genes Measured	DE	%DE
***S. cerevisiae *vs. *S. paradoxus***	0.231	4,362	834	19%	2,688	498	19%

***S. cerevisiae *vs. *S. mikatae***	0.394	3,562	719	20%	2,688	555	21%

***S. cerevisiae *vs. *S. bayanus***	0.556	3,632	806	22%	2,688	609	23%

***S. paradoxus *vs. *S. mikatae***	0.359	3,508	787	22%	2,688	604	22%

***S. paradoxus *vs. *S. bayanus***	0.521	3,587	853	24%	2,688	643	24%

***S. mikatae *vs. *S. bayanus***	0.538	3,001	743	25%	2,688	652	24%

The intergenic substitution rate was reported in Kellis et al. (2003). Differential expression is measured at p < 0.01.

### Identifying the Lineage of Changes in Expression

Under a simple model where changes in expression offer selective advantage to a given organism, one would expect the changes in the genome that underlie these adaptations to become fixed in a given lineage. For example, the gene RME1 (YGR044C) is expected to have higher expression in this strain of *S. cerevisiae *relative to the other three species because there is a known variation in RME1's transcription factor binding site that leads to an increase in the gene's expression [21]. This mutation occurs only in the *S. cerevisiae *genome and is known in one strain to cause an increase in *S. cerevisiae's *sporulation efficiency. In our data, this gene had significantly higher expression in *S. cerevisiae *versus each of the three other species, with fold changes greater than 15X (Additional File 1, Figure S9A). By contrast, the gene TSC3/YBR058C-A had higher expression in *S. cerevisiae *than in *S. bayanus *(p < 0.01) or *S. paradoxus *(p < 0.05), but was not significantly DE against *S. mikatae *(p > 0.05) (Additional File 1, Figure S9B). Differences such as this cannot be ascribed to an individual lineage. While these changes could result from multiple selective events, they are more consistent with a model of drift where changes in expression occur randomly, and do not become fixed in any lineage because they have neutral or nearly neutral phenotypic effects.

**Figure 3:**
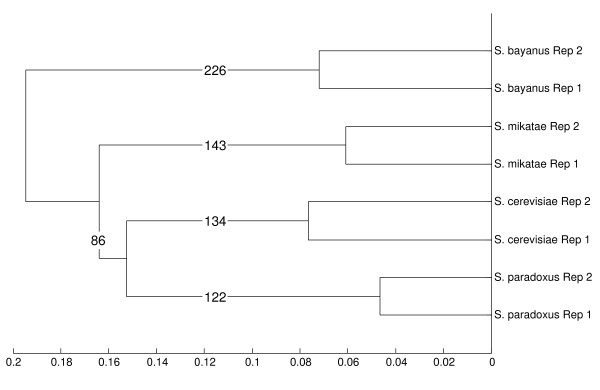
**Hierarchical clustering of the expression of the core genes for the eight samples**. The distance between the samples is 1-the Spearman correlation between the samples. Numbers on the branches represent the number of genes that were differentially expressed in each lineage with evolutionarily consistent relationships, e.g. 226 genes were differentially expressed in the *S. bayanus *lineage vs. each other lineage.

Where possible, we identified the lineage where a change in expression took place (Figure 3 and Additional File 1, Table S5A). We did this using a conservative approach that identified core genes where there was a change in expression that showed a consistent, statistically significant relationship against all of the other species in the lineage.

While a large number of genes were DE in individual cross-species comparisons, only a small number of these differences could be attributed to changes in an individual lineage. For example, 1051 of the core genes (39%) are DE at p < 0.01 in at least one cross species comparison with *S. cerevisiae*, but only 13% of those (134) maintained the DE relationship versus each of the three other branches of the phylogeny. Across the entire phylogeny of these four species, 613 genes showed consistent phylogenetic relationships at p < 0.01 (Additional File 1, Table S5A), and the number of genes diverged at each branch is shown in Figure 3. Overall, lineage-specific changes were equally likely to increase or decrease a gene's expression. Within the individual branches, genes that were differentially expressed in the *S. bayanus *branch were more likely to have decreased expression (p < 0.01 after Bonferroni correction). Ninety-five of the total 613 genes (15%) showed statistically significant changes in more than one lineage. For example, the gene YDL124W showed significantly increased expression in *S. cerevisiae *versus the 3 other species, and significantly decreased expression in *S. bayanus*. Gene ontology analysis revealed no common themes uniting these 95 genes.

While this set of 613 genes showed statistically significant changes, there were few changes of as large a magnitude as the change observed in RME1. Only 10 genes showed changes that were at least 10X versus each of the other species (Additional File 1, Table S5B).

We performed a Gene Ontology enrichment analysis on genes that showed evidence of lineage-specific differential expression to search for evidence of directional selection using AmiGO through the Saccharomyces Genome Database [22]. We used the entire set of core genes as our background set. We found that genes that had decreased expression in the branch separating *S. cerevisiae *and *S. paradoxus *from *S. mikatae *and *S. bayanus *were enriched for genes producing products that localize to the mitochondria (p < 0.01). The genes that had decreased expression in S. mikatae also showed enrichment in the form of three genes that participate in nucleobase-containing compound transmembrane transporter activity in (p < .01). Overall, however, only 25 (4%) of the total 613 genes that had lineage-specific differential expression were members of these enriched GO categories (Additional File 1, Table 5A). No categories were enriched in the other branches.

Overall, these results are consistent with a model where the primary driver of evolutionary changes in expression is random drift, rather than functional selection.

### Expression levels recapitulate phylogeny

**Figure 4:**
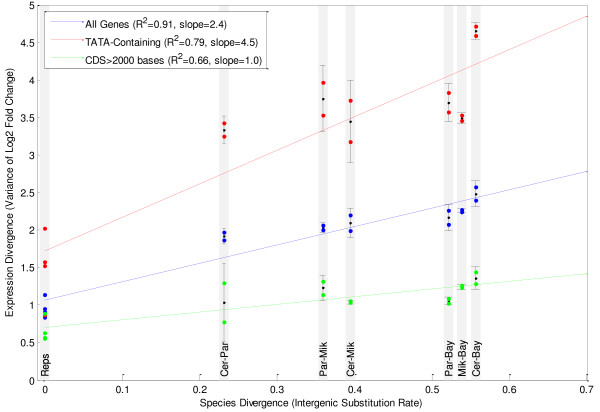
**Linear relationship between variance of distribution of log2 fold changes and intergenic substitution rate between species**. Dots represent the measurements of expression. Error bars are the standard error of the mean.

Gene expression evolves through changes in the recruitment of the polymerase by transcription factors, from which it is reasonable to expect that expression evolves multiplicatively. If this expression evolution were to follow clock-like behavior, then we would expect the σ^2 ^of the log2 fold changes to scale linearly with divergence time between species. To determine whether clock-like divergence is a good model for our expression data, we compared the σ^2 ^of the log2 fold changes to the inter-genic substitution rate between species as measured in [15]. We plotted the σ^2 ^of the log2 FCs for each biological replicate and each same-day species-to-species comparison versus the evolutionary distance between samples. The results showed a close fit to a linear relationship with an R^2 ^of 0.90 (Figure 4). We found similar results using hierarchical clustering on the expression data from each species using the core genes (Figure 3 and Additional File 1, Supplemental Methods 5).

### Classes of genes evolve at different speeds

We examined the rate of evolution between specific classes of genes, based on the variance of the log2 FC distributions for the genes and found significant differences in the rate of evolution between groups of genes.

Genes with a TATA box in the promoter (n = 460, p = 0.016) [23] and genes with a coding sequence shorter than 2000 bases (n = 499, p < 0.001) have diverged more quickly than TATA-less genes and genes with a longer coding sequence. By controlling for each of these factors we observed the increased expression divergence observed in genes with TATA boxes and genes with short coding sequences appear to be independent effects (Figure 4 and Additional File 1, Supplemental Methods 6). The finding that genes with a TATA box diverge faster was previously reported in Tirosh et al. [4] using a dataset of microarray-based expression measurements in four yeast species. We used this dataset to confirm the finding that genes with shorter coding sequences diverge faster (p < 0.001), though the correlation between length and expression divergence is weak (-0.23).

Genes with more than 5 transcription factor binding sites [24] and genes whose mRNA decays more slowly than the median [25] also had significantly higher rates of expression divergence (p < 0.01). However, both of these properties are more common in genes with TATA boxes, and we could not demonstrate that this effect is independent of TATA status. When we controlled for the number of transcription factor binding sites and mRNA decay rates, genes with a TATA box still had an increased rate of expression divergence, indicating that neither of these factors is responsible for the behavior of genes that contain a TATA box.

Potential confounding factors such as the expression level of the gene (p = 0.99), the variance of the gene in biological replicates (p = 0.85), and mean coding sequence divergence (p = 0.60) were not significant components of expression evolution in these species (Additional File 1, Supplemental Methods 6).

While genes with TATA boxes and shorter coding sequences diverged faster than non-TATA genes and genes with longer coding sequences, the σ^2 ^of these classes still maintained a linear relationship with regards to divergence time (R^2 ^of genes with TATA boxes = 0.80, R^2 ^genes with CDS > 2000 bases = 0.66, R^2 ^of genes with CDS < 2000 bases = 0.86 Figure 4).

### Gene duplication events often give rise to an heir and a spare

We examined whether having duplicated copies of a gene increased the total expression of the gene following a gene duplication event. To do this, we tested whether we could detect differential expression between the copies of duplicated genes following what appeared to be lineage-specific duplication in *S. bayanus*, the species most diverged from *S. cerevisiae*. While there are many *S. cerevisiae *genes annotated with non-unique *S. bayanus *orthologs [15], we used additional filtering to ensure that the gene was a duplication that could be accurately assessed with RNA-Seq. These criteria included ensuring that each copy of the gene in *S. bayanus *covered more than 60% of the original the *S. cerevisiae *genes, which excluded most potential duplication events (Additional File 1, Supplemental Methods 7 and Table S6). Filtering left us with a set of 14 stringent gene duplications.

**Figure 5:**
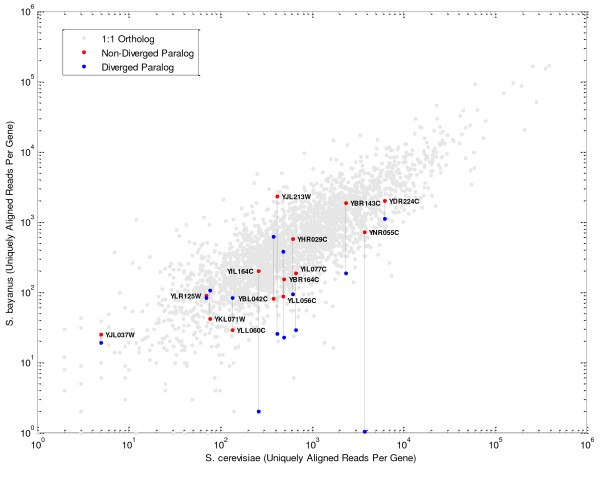
**Expression of paralogs following likely gene duplication events in *S. bayanus***. Both paralogs are plotted vs. the expression of the single *S. cerevisiae *ortholog. Gray points show the expression of S. *bayanus *vs. S. *cerevisiae *1:1 orthologs.

For each pair of paralogs, we determined which gene sequence was more diverged from the common ancestor by creating a phylogenetic tree of the orthologous sequences from all species. In 10 out of the 14 cases, the less divergent copy maintained an expression level consistent with *S. cerevisiae *while the divergent copy dropped in expression level (Figure 5). In 5 of these cases, the diverged paralog had an expression significantly below the level of expression observed in *S. cerevisiae*. There were no examples where the less divergent copy dropped expression to this degree. Additionally, there were no examples where expression dropped substantially for both duplicated genes. Overall, this suggests a predominant model where one of the duplicates inherits the function of the original gene while the other becomes obsolete.

## Discussion

RNA-Seq offers a significant improvement in measuring cross-species gene expression over microarrays because it allows for orthologous genes from multiple species to be measured and compared to one another without the significant complications of species or strain-specific probe biases. For this reason, it is likely that RNA-Seq will replace microarrays in cross-species experiments. However, many of the challenges that were present in measuring gene expression using microarrays will continue to be present in RNA-Seq experiments because they originate from biological rather than technical sources. Overcoming these challenges in cross-species experiments will be particularly difficult because measurement artifacts can both mimic and obscure the effects of evolution. For example, we found that shorter genes tended to have higher expression divergence between species and are more likely to be differentially expressed. This finding unlikely to be an artifact of RNA-Seq measurements as it is consistent with a finding in a microarray-based study of drosophila [6], and was observed in the microarray measurements of yeast [4]. However, shorter genes that are truly DE are less likely to be detected as such than longer genes with expression changes of the same fold change because their transcripts produce fewer nucleotide fragments, and thus are measured relatively higher Poisson noise.

Normalizing read counts between samples can also be problematic. Under a neutral model of gene expression evolution, the number of genes with increased expression in one species versus another should equal the number with decreased expression. Deviations from symmetry can be viewed as evidence of selection [26]. Most methods for normalizing RNA-Seq data assume that the overall expression of the two species will be the same, forcing some degree of symmetry. Because reference assemblies will usually differ in quality in cross-species experiments, it is important to use normalization strategies that are robust to the effects of missing regions and errors in genomes. Missing regions will have a limited effect on our normalization method because only genes that are present in both species are included in the normalization equations. The effect of errors can be better ameliorated by using an alignment strategy such as ours that allows for some mismatches between the reads and the reference genome. In our qPCR validation, our results did not show any systematic, directional differences compared to our RNA-Seq results. Despite this strategy, we did see a tendency for genes with consistent expression changes in *S. bayanus *to have decreased expression. There was, however, no evidence that these genes represented a functional class of genes, and across the entire phylogeny genes with changes in expression were equally likely to have increased and decreased expression.

To confirm that our statistical approach adequately accounted for both technical and environmental variance in our experiment, we validated our DE calls using qPCR with RNA isolated from biological replicates grown separately from the samples used for RNA-Seq. By contrast, running qPCR on the same RNA used for RNA-Seq would have only assessed whether the technical variance was correctly assessed. RNA-Seq measurements are already known to have a high level of technical reproducibility, in our study and others [12,13], and therefore validations of technical reproducibility add little value. Because our qPCR was run on independent samples, and our results generally reproduced within our calculated confidence, we are confident that significant p-values correspond to the probability of consistent results if the experiment were repeated.

The sensitivity of experiments to detect differentially expressed genes is determined by the ability to distinguish true changes in gene expression from alternate sources of variance. Sensitivity in RNA-Seq experiments will be limited for genes that are measured with low read counts and high Poisson counting noise. In this experiment, our samples were sequenced deeply enough that we could measure the expression of greater than 95% of genes with greater than 10 reads. This sequencing depth is much more difficult to achieve in mammals and other species with large transcriptomes. We found that for genes measured with fewer than 10 reads, the signals from biological and technical variance were largely obscured by Poisson sampling noise, and only large fold changes could be detected as significant. While deeper sequencing will reduce this Poisson noise, using the same number of reads to sequence an additional replicate will reduce both Poisson and non-Poisson variance, and should theoretically provide a greater increase in sensitivity than deeper sequencing.

In a review of studies of gene expression evolution, Clarke et al. [27] noted that the reported DE rates between evolutionarily diverged organisms shows a high degree of variation between studies, and suggested that this variation was caused by differences in the sensitivity of the experiments. Additionally, the way in which biological variance is measured and accounted for in the experimental design is a crucial factor in determining not just how many genes will be detected as DE, but also the reproducibility of findings. For example, Cassone et al. [28] reported that the calculated rate of DE genes between two insipient species of mosquitoes was 1-2% of genes when they applied a model that correctly controlled for the biological variance observed between colonies of mosquitoes, but would be measured at ^~^54% if less stringent controls were applied. Their finding of little differential gene expression in these closely related strains is consistent with our results.

Large deviations from our linear model of expression divergence would be possible if a single mutation altered a gene's expression dramatically, and this event had a cascading effect on the expression of other genes. However, we believe that in wild populations such changes are likely to be selected against. For example, the change in expression of RME1 demonstrates that a single base mutation can dramatically alter the expression of a gene. However, despite the high divergence within the intergenic regions of these species, there were only ten examples of changes in expression that were of the same magnitude as the change observed in RME1. A role for purifying selection is further supported by our finding that the more diverged copy of duplicated genes also dropped in expression 35% of the time while there were no such cases for the less diverged copy. This finding is consistent with, and further clarifies, microarray-based findings of expression divergence following gene duplication events [29,30].

While there are clear signs of purifying selection in our study, several lines of evidence support the premise that the primary driver of changes in expression is neutral drift. Only a small fraction of the genes differentially expressed between individual species showed evidence of being fixed in any lineage. While two Gene Ontology categories were over-represented in genes exhibiting lineage-specific DE, the majority of such genes were not members of these categories. Furthermore, the linear correlation between the expression divergence and the intergenic substitution rate suggests a clock-like divergence. While properties such as TATA boxes and gene length were associated with increased gene expression divergence, divergence rates within these categories still maintained a linear relationship with intergenic substitution rate, consistent with a drift model with a faster diffusion coefficient.

A recent study of a *S. cerevisiae - S. bayanus *hybrid identified several categories of genes as being directionally differentially regulated between *S. cerevisiae *and *S. bayanus*, and suggested that these categories were under evolutionary selection [31]. We did not replicate differential expression in any of their Gene Ontology categories in our study. There were, however, large differences between our study designs and the statistical analyses used. A hybrid construct is used to identify genes that are expressed at different levels due to differences in transcriptional efficiency caused by cis regulatory differences. In Tirosh et al. [32] it was shown that genes observed as DE between species are not necessarily observed as DE within the hybrid, and vice versa. Additionally, our strains were prototrophic, whereas the hybrid strain had several auxotrophies and therefore required amino acid supplements in the media, making the growth conditions in the two experiments different. The auxotrophies may be particularly important differences in light of the fact that two of the five networks that Bullard et al. identified as directionally regulated in the hybrid (histidine and lysine biosynthesis) contained pathways that were not functioning normally because the strain was auxotrophic for his3 and lys2.

## Conclusions

Overall, we found that the changes in expression that occurred in response to even the subtle environmental variation of a well-controlled laboratory were similar in magnitude to the changes in expression that accumulate over millions of years of evolution. This finding emphasizes the need to design experiments that measure the evolution of gene expression to avoid introducing any alternate sources of expression variation into the experiments. The statistical significance of gene expression changes measured in RNA-Seq experiments must account for the effects of these different types of variance in each measurement.

## Methods

### Samples Preparation

All cultures were grown to saturation overnight in 25 mL of SD (6.7 g yeast nitrogen base, without amino acids, Difco# 0919-15 + 2% glucose). This was then diluted ^~^1:100 into 1 L of prewarmed SD in 2.8 L flasks at 30C shaking at 240 RPM. Cells were grown to an OD600 of 0.8-0.9. We had previously measured the growth profile for all four of the strains we used by counting cell number over time (with a hemocytometer) and comparing this to OD. From this we determined that all four strains were still in exponential growth at this OD. Ice was added directly to the cultures to stop growth. The cells were spun down at 3000 g for 5 minutes. The pellet was washed 1x with 25 mL of ice cold water and transferred to a 50 mL falcon tubes. The cells were spun 3000 g for 5 minutes and washed one more time in ice-cold water. After the final wash the cells were resuspended in 1 mL of ice cold water and aliquoted to 5 tubes. The cells were spun down at top speed on a table top centrifuge. The liquid was removed and the cell frozen in liquid nitrogen. The experiments were performed in duplicate. All steps were also duplicated. An independent isolate of each strain was taken from a plate, and media was media separately (not a single batch for all experiments).

### RNA Isolation and Sequencing

Whole transcriptome RNA-Seq (WT-Seq) was performed according to a protocol/kit now available from Life Technologies, with minor modifications that are described below. Briefly, 5-10 μg of RNA isolated from each of the eight yeast cultures was depleted of ribosomal RNAs using two rounds of Eukaryotic RiboMinus treatment (Life Technologies) with overnight ethanol precipitations for sample re-concentration. The removal of ribosomal RNAs was confirmed on a Bioanalyser Nano Chip (Agilent). A total of 500-1,000 ng of riboRNA-depleted total RNA was fragmented for 18 min at 95°C in NEB buffer 3 followed by 30 minutes PNK treatment in the same buffer. Fragmentation was followed by size selection of ^~^50 to ^~^150 bp fragments using the flashPAGE denaturing PAGE-fractionator (Life Technologies) and ethanol precipitation overnight. The resulting RNA was directionally ligated, reverse-transcribed and RNaseH treated.

After trial PCR to assess library quality and quantity, 30 μl cDNA was run on a native 6% PAGE gel. The 90-120-bp size window (corresponding to 50-80-bp RNA insert size) was cut from the gel, shredded and inserted directly into a 400 μl PCR reaction using standard WT-Seq kit components and submitted to 11-15 cycles of PCR. The PCR product was phenol-chloroform extracted, ethanol precipitated and re-suspended in 20 μl WT-Seq gel loading buffer. The resulting sample was run on a 6% native PAGE gel, and the 150-175-bp size range (corresponding to 60-85 bp) was cut from the gel, shredded, and extracted overnight in WT-Seq PAGE elution buffer. The resulting library was filtered through 0.45 μm spin filters (Life Technologies) to remove gel pieces and ethanol precipitated.

We note that WT-Seq can detect neither the 5'-most fragment from transcripts with 5'-modified ends (such as mRNA 5' 7-methyl-guanosine caps) nor the 3'-most fragment from transcripts with 3'-modified ends. However, for transcripts long enough to produce multiple ≥ 50-bp fragments, WT-Seq should detect the remaining fragments.

### Alignment and Quantification

Reads were aligned to their respective genomes using the MOSAIK alignment program allowing for a threshold of two mismatches between each 35 base pair read and the reference genome. The alignment output was parsed using the bamtools API [33] and custom procedures written in C++ and Matlab.

As our main unit of quantification, we used the number of reads uniquely aligning to an annotated region of the genome (usually coding sequence). Several other groups have normalized read counts by the length of the gene that is unique enough for reads to align to calculate RPKM [34]. We did not do this because we wished to preserve the raw count of the reads in order to assess the amount of Poisson noise that is present in a gene's measurement when we performed our statistical analyses.

### Inclusion Criteria for Measured 1:1 Orthologs and Core Genes

Genes that were selected for inclusion in the comparison of expression of 1:1 orthologs met the following criteria: the gene was annotated in *S. cerevisiae*; in non- *S. cerevisiae *species it was annotated with one ortholog [15]; its annotation was for a complete ORF (i.e. the ORF did not extend into an unfinished part of the draft genome); the annotated length was within 90% to 110% of the *S. cerevisiae *annotated length; and, for each sample where the gene was measured, the number of uniquely aligned reads for this gene was > = 90% of the total aligned reads.

Core genes met the above criteria for all four species (Additional File 1, Table S7). The purpose of this set is to be able to make species-to-species comparisons using the same gene set in all four species. Because we required that genes be conserved in all four species, this set will contain only conserved genes. However, genes that are missing from one of the non-*S. cerevisiae *lineages are more likely to be in unfinished portions of their draft genomes rather than actually missing in the species. All core genes had expression detected by at least one read in one species, and all but 10 genes were expressed at detectable levels in all four species.

### The Χ^2 ^Test Statistic

We assessed the statistical significance of differential expression using a two-by-one Χ^2 ^test to test the hypothesis that a gene is expressed at the same level in both species [35]. If a set of N independent samples (x) are randomly drawn from a normal distribution of a known mean (μ) and variance (σ^2^), the resulting set of test statistics, as calculated by Equation 1, will form a *χ*^2 ^distribution with N degrees of freedom [36]:

Equation 1

$${\text{X}}^{2}=\sum \frac{{\left(x-\mu \right)}^{2}}{\sigma^{2}}$$

This relationship can then be used to calculate the probability that the expression level for the gene in the control (C_g_) and the test (T_g_) conditions were drawn from the same normal distribution. The principle challenges are to correctly calculate the mean of the common distribution and its variance.

### Calculating the means of the common distribution with normalization

Even when read counts for the same gene are drawn from the same underlying distribution, the mean of this distribution will be measured at a higher or lower value depending on how deeply each sample is sequenced. Separate means (μ) must be calculated for the control and test condition that are proportional to the sequencing depth of the sample. Several papers have reported on the importance of normalizing the RNA-Seq read counts by the appropriate factors to account for differences in the total number of reads sampled in the two datasets, as well as the underlying mixture of RNA in the samples [37]. The principle challenge is that a low number of highly expressed genes with outlier measurements can significantly affect the total read count for one of the samples, and the ratio of total read counts in both samples is therefore not a reliable metric to use to normalize samples.

We visualized the problem by plotting simulated raw read counts (See Additional File 1, Supplemental Methods 4 for simulation procedure) on a scatter plot with the counts for the genes in the control sample on the × axis and the test values on the Y axis. We then found the slope (m) of the line that most closely matched the known, simulated slope was calculated as the median ratio of test to control counts, a metric which has been used previously [38] (Additional File 1, Figure S10 and Supplemental Methods 8).

The means of the distributions for each of the two samples are points along this line. The exact point is found by taking the midpoint of the segment of the slope line that is bounded by (C_g_, C_g_m) and (T_g_/m, T_g_), as shown in Additional File 1, Figure S11. This point gives an unbiased weighting to both the control and the test condition, even when one sample is sequenced much more deeply than the other, and allows for the calculation of a distribution mean for each sample without rescaling the read counts.

### Calculation of Variance

Because RNA Seq experiments commonly use a low number of replicates, care must be taken to ensure that accurate variance estimates are used. Because Poisson and non-Poisson variance are independent from one another (Additional File 1, Figure S12), total variance can be calculated by adding the independent components of the variance [39]. Because Poisson variance is dependent upon sequencing depth, while non-Poisson variance is not, it is useful to separate out these two components in order to ensure a more accurate calculation of the variance.

We identified two approaches which can be used to estimate the non-Poisson variance. The first, simply measuring the variance in gene-specific read counts across replicates, provides an unbiased estimate of variance. The variance measured in replicates for a given gene (σ_reps_^2^) will include the combined effects of Poisson and non-Poisson variance (*σ_nP_*). Poisson variance can be estimated as the mean of the number of reads that were used to make the measurement of variance in replicates (R), and separated from non-Poisson variance as follows:

Equation 2:

$${\sigma_{nP}}^{2}={\sigma_{reps}}^{2}-R$$

The measured gene-specific relative non-Poisson standard deviation (σ_m_) is found by dividing the non-Poisson variance by the read count:

Equation 3:

$$\sigma_{m}=\frac{\sigma_{nP}}{R}$$

And is thus:

Equation 4:

$$\sigma_{m}=\frac{\sqrt{{\sigma_{reps}}^{2}-R}}{R}$$

The accuracy of the measured variance as an estimate of the variance of the normal distribution from which the sample is drawn will be limited by the number of replicates used and by the amount of Poisson noise that is present in the measurements that were used to estimate the variance. For example, we do not believe that expression levels can be measured with complete certainty using RNA-Seq because the values in the technical replicates do not repeat with 100% reliability (Additional File 1, Figure S1). However, some of the technical replicate variances (*σ_reps_*^2^) will be measured at zero because the measurements will repeat exactly in the both replicates by random chance. Error in the estimates of non-Poisson variance will be highest for genes which are measured with few reads. This is because the variability in measurements across replicates will be dominated by Poisson noise making estimates of non-Poisson variance unreliable. Despite these drawbacks, the measured variance is still the most unbiased approximation of true gene-specific variance.

An alternative approach is to assume a uniform over-dispersion, where all genes are assumed to have the same non-Poisson variance which is set to a uniform over-dispersion factor (U). The value used as the uniform over-dispersion (U) can be calibrated based on replicates. By definition, the p-value is the probability that a difference will be detected as statistically significant (rejection of the null hypothesis) when in fact no difference exists. In replicates, the null hypothesis is true for all genes, but would be expected to be falsely rejected in proportions equivalent to the calculated p-values. The U is therefore set as the minimal U such that the percentage of genes that are called DE in biological replicates equals the p-value. For example, if 10,000 genes are calibrated to a U with a p-value of 0.05, the U is the minimum value where 500 of the genes in the biological replicates have a p-value < 0.05. The calibration was performed on genes with a mean count of at least 10 reads across the two conditions. This cut-off ensures that measurements are not dominated by Poisson noise which may skew calibrations. The value of U will be approximately the center of the distribution of measured non-Poisson variances. The disadvantage to using a uniform over-dispersion is that it corrects all variances towards a central value. This correction systematically underestimates the variance of classes of genes which have high variance, while overestimating the variance for low variance genes. This correction can therefore introduce biases into differential expression calls.

We resolve these two approaches by further breaking non-Poisson variance into a technical variance and gene-specific environmental variance. Technical variance is caused by errors in the library preparation and sequencing process. While it is probable that some coding genes may be more susceptible to technical variance than others, we do not have sufficient information to identify these genes and therefore our best estimate of variance is a uniform model where each measurement is assumed to have a minimal possible *σ_nP _*equal to technical variance (*σ_t_*). This minimum *σ_nP _*is effectively the resolution of the RNA-Seq technology. Genes should not be called DE between two samples if they are closer in expression than the resolution of the technology. To assess this resolution we calculate the U for our technical replicates using coding genes (*σ_t _*= 0.16 in our experiment).

Gene-specific environmental variance cannot be assumed to be uniform because a gene's response to the environment is determined by its function. Using a uniform model to assess this variance will yield false positives that are enriched for genes which have high variance in biological replicates. This is undesirable because it is difficult to distinguish such artifacts from biological effects. Therefore, when the measured gene-specific variance *σ_m _*was above the uniform technical variance, this value was used as the estimate of *σ_nP_*:

Equation 5:

$$\sigma_{nP}=\max\left(\sigma_{m},\sigma_{t}\right)$$

To improve the accuracy of our calculations of variance, we assumed that the gene-specific variance will be constant across all four species because gene function is likely to be conserved. This assumption will give each gene a greater number of data points on which to base the estimate of variance. We then took the weighted mean of the non-Poisson relative variances that were calculated for each gene across the biological replicates of the four species. Measurements were weighted by the number of reads on which they were based under the assumption that genes measured with more reads will have more accurate assessments of variance because they will be subject to less Poisson noise. Measurements made with less than a mean of 30 reads in the two replicates were discarded on the assumption that they were unreliable due to the fact that more than 20% of their measured variance is Poisson noise. When the gene was not measured by at least 30 reads in any of the species its variance was set to U calibrated for that species' biological replicates. Because our experiment had sufficient sampling depth, this correction applied to only 3% of the core genes.

Because each species' replicate pair reproduced with a different overall variance (U), when the measured gene-specific variances are used in the Χ^2 ^test statistics they are scaled to reflect these small differences by multiplying by the U calibrated for the species divided by the mean U across all four species.

### Degrees of Freedom

Each Χ^2 ^test statistic was calculated with an N of 2 (the control condition and the test condition), which would suggest that the test statistic should have 2 degrees of freedom. However, the means of the sampling distributions were calculated based on the measurements from both samples. For this reason, a degree of freedom is lost. To demonstrate this, we show that a sampling distribution of Χ^2 ^test statistics generated under the null model using the measured non-Poisson variance (*σ_nPC _*= *σ_m_*) is reasonably well approximated by a Χ^2 ^distribution with 1 DF (Additional File 1, Figure S13A). Using a uniform variance in the Χ^2 ^test statistic (*σ_nPC _*= *U*) also produces a Χ^2 ^distribution with one degree of freedom (Additional File 1, Figure S13B).

The corrections to the Χ^2 ^test statistic are designed to use all of the information available in the experiment to provide the most accurate identification of DE genes without introducing substantial biological biases in the genes that would be falsely detected as DE. In some cases where the total variance calculated from the replicates was lower than the inherent technical variance calculated from Poisson fluctuation plus the over-dispersion between technical replicates we used the technical variance measured in replicates to represent the truth. Because of this correction, the final distribution of Χ^2 ^test statistics under the null model shows a slight deviation from the Χ^2 ^distribution with 1 DF, indicating that in some cases our p-values may be slightly conservative (Additional File 1, Figure S13C). In biological replicates, the false positive rate at p < 0.05 was 3%.

### Combining tests

We found that our fold change (FC) calls reproduced better when we only compared samples which were prepared on the same day (Additional File 1, Supplemental Methods 9 and Table S8). This finding indicated that there are batch effects within the data that must be controlled for. Therefore, we performed two tests for each cross-species comparison: Rep 1 versus Rep 1 and Rep2 vs. Rep 2. We treated each test as an independent test and combined p-values using Fisher's combined probability method [40].

### Calculation of Confidence Intervals Around Fold Changes

The fold-change was calculated as T_g(n)_/C_g(n) _where T_g(n) _and C_g(n) _are the read counts, normalized by sample size, for each gene in the test and control samples (Additional File 1, Supplemental Methods 10). The confidence interval around the fold-change is calculated as follows:

Equation 6:

$$\sigma_{\underset{\bar{C}}{F_{T}}=\sqrt{{\left(\frac{\sigma_{Tg}}{T_{g}\times \text{log(2)}}\right)}^{2}+{\left(\frac{\sigma_{Cg}}{C_{g}\times \text{log(2)}}\right)}^{2}}}$$

This confidence interval is attained by propagating the uncertainty in each measurement [39]. See Additional File 1, Supplemental Methods 10 for derivation.

### Measuring the rate of expression divergence

To measure the rate of expression divergence, we used least squares regression to draw the line of best fit through the σ^2 ^of the log2 FCs plotted against the intergenic substitution rate. The slope of the line is the measurement of the rate of gene expression divergence, while the Y intercept is the variance of the log2 fold changes at time = 0 (biological replicates). The Y intercept is therefore higher for subsets of genes with larger variance in replicates. We tested the significance of slope differences using an ANOCOVA test. To eliminate the possibility that these results were artifacts of the RNA-Seq technique, where possible, we verified our results against the data of Tirosh et. al [4], an experiment that used microarrays to calculate expression response divergence at the individual gene level in genes under different stress conditions in *S. cerevisiae, S. paradoxus, S. mikatae*, and *S. kudriavzevii *(Additional File 1, Supplemental Methods 6).

## List of abbreviations used

CDS: coding sequence; DE: differentially expressed; FC: fold change

## Authors' contributions

MB participated in the study design, performed the main bioinformatics analysis, and drafted the manuscript. JG conceived of the study and participated in study design and analysis. JG and AC developed the protocol and built the sequencing libraries. CS provided guidance on the statistical analysis and helped to draft the manuscript. MPS and DB provided code to perform the alignment and reads processing. JC advised on the analysis. MS grew the yeast strains and participated in the study design and analysis. GM participated in the experimental design, coordinated, and oversaw the project. All authors read and approved the final manuscript.

## Supplementary Material

Additional file 1 **This file contains Supplemental Methods and Supplemental Figures**. Click here for file

Additional file 2 **This file provides the output for each cross-species orthologus gene comparison, including the counts of unique reads in each species and the results of the X^2 ^statistical test**. Click here for file
