# Supplementary material for: Expression divergence measured by transcriptome sequencing of four yeast species
Source: BMC Genomics. 2011 Dec 29;12:635. doi: 10.1186/1471-2164-12-635 (PMC3296765; doi:10.1186/1471-2164-12-635)
Supplement: Additional file 1 — This file contains Supplemental Methods and Supplemental Figures. [file 1471-2164-12-635-S1.PDF]

Expression Divergence Measured by Transcription Sequencing of Four Yeast Species: Supplemental Methods and Figures

Supplemental Methods

1. Reference assemblies, annotations, orthology mappings, and alignment
2. The dominant source of unaligned reads is sequencing errors.
3. qPCR
4. Data simulation
5. Clustering and bootstrapping to prevent reuse of data points
6. Classes of genes with faster divergence
7. Finding measurable gene duplication events
8. Normalizing data
9. Measurements taken on the same day reproduced better
10. Calculation of fold change and the derivation of confidence interval around the log fold change

Supplemental Figures

1. Technical versus biological replicates
2. Contribution of each source of variance to total measurement uncertainty
3. Estimated fold change equivalency between microarrays and RNA-Seq
4. Comparison of fold changes in biological replicates vs Nagalakshmi
5. Batch Effects
6. Comparison of fold changes from RNA-seq versus qPCR
7. Performance of our modified chi-square vs. DESeq and a t-test on simulated data
8. Differential expression in pairwise comparison
9. Changes in expression in RME1 and TSC3
10. Calculated versus actual slopes using various normalization methods
11. Calculation of expected values (means) in our modified chi square.
12. Poisson and non-Poisson variance are uncorrelated
13. Fit of sampling distributions to a X^2 distribution with one degree of freedom.
14. Fit of parameters to mathematical distributions
15. Simulation results

Supplemental Tables

1. Alignment results
2. Fold changes observed for the same gene between measurements in biological vs. technical replicates
3. qPCR measurements
4. Comparison of genes called differentially expressed by methods
5. Genes differentially expressed in each branch of the phylogeny
6. The number of genes remaining after each inclusion criteria for potentially gene duplication events
7. Measured genes and the reasons for gene exclusion by species
8. The number of genes replicating within observed confidence intervals in same day versus different day comparisons

Supplemental Methods

1. Reference assemblies, annotations, orthology mappings, and alignment

The *S. cerevisiae* genome and annotation were downloaded from the "Saccharomyces Genome Database" http://downloads.yeastgenome.org/ on 1/28/2010. S. *paradoxus, S. mikatae,* and S. *bayanus* genome sequences and annotations were published by Kellis et al. [1] . Annotations of these three species were made based on orthology mappings relative to *S. cerevisiae*. The ortholog mappings in [1] were used in all comparative analyses. Kellis et al. reported the genome sequences as contigs, but included a mapping of how these contigs mapped onto scaffolds. Because these contigs sometimes overlapped, we assembled the contigs onto scaffolds prior to alignment and transposed the annotation positions onto the scaffold positions.

For differential expression of 1:1 orthologs, the MOSAIK parameters were set so that reads that aligned to the genome with 2 or fewer mismatches across their 35 base pair length were aligned. When analyzing paralogs, we used the more restrictive setting of 1 mismatch to obtain better discrimination between paralogs. The alignments included reads which aligned to only one position (uniquely aligned) and reads which aligned to many positions (non-uniquely aligned). Allowing for mismatches compensates for potential single nucleotide variations in the samples relative to the reference, and the fact that the three other genomes are not as deeply sequenced as S. *cerevisiae* and their reference assemblies are expected to contain more errors.

2. The dominant source of unaligned reads is sequencing errors.

We achieve a 54% alignment rate for our reads. The dominant source of unaligned reads in our experiment was overwhelmingly errors in the reads themselves. We established this by filtering reads to only include the highest quality reads, where we were confident that there was a very low chance of sequencing error, and aligning only these reads to the genomes. When we eliminated read errors as a source of unaligned reads, we achieved a 92-98% alignment rates for each sample.

Our filtering strategy was as follows: The AB Pipeline produces a large number of reads but does not stringently filter them based on quality. However, each di-nucleotide transition call in each read comes with an associated transition quality score, which is analogous to the PHRED base quality score used in other technologies. These scores can be approximately transformed to the probability that the transition call is correct. If we assume each probability is independent, multiplying each of the transition scores sequentially gives the probability that entire read has no errors:

$$probablity\ read\ correct = \prod_{i=l} 1 - 10^{\frac{q_i}{-10}}$$

where *l* is the length of the read (36), *i* is the position in the read, and q_i is the transition quality at position *i*.

For example, if a three transition read has qualities 30, 20, and 10, then the probability it is correct is:

0.9997*0.99*0.9=0.89

Often the quality scores provided by manufactures need to be recalibrated slightly to ensure accurate probabilities, e.g. [2]. However, while this probability score is only an approximation, it is still an excellent and quick way of identifying what reads are least likely to contain errors.

We filtered reads based on quality scores to create a subset of high quality reads which have at least a 95% probability of having only correct calls in the read. The alignment rate for the high-quality reads ranged from 92% to 98% (Supplemental Table S1). A substantial portion of the unaligned reads from the high-quality read alignments corresponded to primer sequences that were sequenced. While alignment rates did decrease as reference assemblies became less finished, the alignment rates for the species with draft genomes were very close to the alignment rates for the S. *cerevisiae* samples, indicating that the unfinished state of the draft genomes caused only a minor reduction in the overall alignment rates for these species (Supplemental Table S1). Reads crossing splice junctions also did not appear to make up a significant portion of the reads, as would be expected in these species which perform minimal splicing. When using any pipeline that does not stringently filter reads, we recommend filtering and aligning reads as an early quality step to ensure that there are no obvious problems in the experimental data (e.g. sample mislabeling, contamination).

3. **qPCR**

We chose 10 genes for qPCR analysis. Genes were chosen to represent the entire dynamic range of expression values. Five genes had large fold changes against S. cerevisiae, and five had small fold changes. One of the large fold change genes, YMR095C (SNO1), was subsequently excluded from further analyses because we observed that it appears to have much heavier antisense transcription in S. *cerevisiae* than in the other three yeast species. qPCR is not strand-specific, convoluting the results.

Confidence intervals were calculated as described in Supplemental Methods 10. In the comparison with DESeq, the values for the mean expression and variance were obtained using the varianceFitDiagnostics tool. The values used for the mean and variance were the baseMean and baseVar.

4. Data Simulation

We used MATLAB to simulate RNA-Seq read count data so that we would be able to assess how our statistical methods performed using data where we knew which genes were DE, and to compare various analysis strategies, for example the X^2 normalization procedure. We simulated four components: 1) The basic expression levels between different genes in the same sample 2) the length of the genes 3) the variance in expression for a single gene in multiple replicates, and 4) counting noise that occurs with sampling. For simplicity, we assumed that all portions of reads could be uniquely aligned to the genome. Most of the distributions of the actual data were well fit to lognormal distributions. These fits are summarized in Supplemental Figure S14.

Expression Level – Transcript Count and Gene Length

We used lognormal distributions to simulate gene expression level (transcripts per gene) and gene length. We multiplied the two values to get an underlying gene expression distribution, which give an approximation of the number of nucleotides that each transcript will generate. In our simulations, we used the mean and standard deviations from the S. *cerevisiae* replicate 2 sample. The use of a lognormal distribution to simulate expression level differs from the more commonly used Zipf's law [3], but provided a better fit for our data, particularly for conserved genes.

Variance Between Samples

We added variation to each transcript count to simulate biological variance and technical measurement imprecision. We first added biological variance. We assumed that each gene would have a gene-specific variance in response to environmental noise, but that this variance would remain constant across the four species. Variance was modeled with a normal distribution and technical variance was modeled using a uniform overdispersion.

Because each gene has an individual variance, we assumed that the measured gene expression would be drawn from some normal distribution with the mean equal to the expression level, and width of the distribution determined by the variance. Genes with more variance would have expression drawn from a wider distribution. After adding biological variance, we then repeated this process to simulate technical variance using the same procedure.

We found no correlation between a gene's non-Poisson variance and its expression level in the data (Supplemental Figure S12), so these effects are simulated independently.

Count Noise

To simulate counting noise, the resulting gene expression values were given Poisson noise. This step most significantly affected genes with low expression.

Different Sample Sizes and Differential Expression

The total counts of mapped reads were also varied between samples to simulate varying sample sizes. Differential expression was modeled by adjusting base expression levels according to an effect size factor (Cohen's D) before variance was modeled.

Simulation Results

Correspondence between the simulated and actual data is shown in Supplemental Figure S15. The simulation can produce technical and biological replicates which can be used for variance calculations.

5. Clustering and Bootstrapping to Prevent Reuse of Data Points

We sought to determine whether the expression levels of orthologous genes would reflect the phylogenetic relationships between the species. To answer this question, we first performed hierarchical clustering on the expression data for each biological replicate from each species using for the core genes. Count values were scaled to the size of the *S. cerevisiae* rep. 1 sample, values were log2 transformed, and the Spearman correlation was used as the distance metric between samples (Euclidean and Pearson correlations gave similar results). Linkage based on the average (UPGMA) distance was used to determine the tree (Figure 3). Biological replicates for each species clustered together as expected, though the distance between the replicates was not negligible compared to the distance between species. The distance between samples was linearly correlated with phylogenetic distance between species (R^2=0.87). Phylogenetic distances were those determined from comparisons of intergenic sequence (Kellis et al. 2003). The strong correlation between expression distance and phylogenetic distance demonstrates that expression data and sequence data as a rule evolve consistently.

Note that the data points from each gene-comparison were used multiple times. For example, the measurements from S. *cerevisiae* replicate 1 were compared to S. *cerevisiae* replicate 1, S. paradoxus replicate 2, S. mikatae replicate 2, and S. bayanus replicate 2. Reusing the same measurements can bias the results. To assure that this was not the case, we performed a bootstrap sampling of the data to avoid reusing the same measurements in different comparisons. This was done by dividing each dataset into three groups based on randomly assigning genes to one or other of the groups, and reanalyzing the data using these subsamples as data points. Each measurement was therefore only used once. The R^2 of this comparison was 0.95.

6. Classes of genes with faster divergence

Genes with a TATA box diverged at a faster rate than non-TATA containing genes, with a slope of 4.5 compared to 2.7 (n=460, p=0.016). The divergence of genes with and without TATA boxes maintained their linear relationship with intergenic substitution rate, having R^2 values of 0.79 and 0.82, respectively.

Genes with a coding sequence longer than 2000 base pairs in S. *cerevisiae* had a significantly lower gene expression divergence than shorter genes (n=499, p<0.001), yet both classes of genes still retained a linear relationship of divergence with intergenic substitution rate (R^2= 0.62 for >2,000, R^2= 0.76 for <2,000). This difference in divergence was still significant when considering only genes with (p<0.01) or without (p<0.001) TATA boxes, and was confirmed among core genes in the Tirosh dataset [4] (p<0.001), though the Pearson correlation between length and expression divergence is weak (-0.23). Having a TATA box significantly increased the expression divergence in genes of all lengths, including sequences longer than 2,000 (p=0.02), 1000-2000 (p<0.001), and shorter than a 1,000 bases (p<0.01), indicating these are independent effects.

Genes with larger regulatory footprints would be expected to diverge in expression more quickly because random SNPs would have a higher chance of hitting a regulatory region of the gene. Genes with multiple transcription factor binding sites (TFs), as annotated by [5] diverged at a faster rate than genes with a single TFs, though this increase only met statistical significance when the genes with 5 or more TFs were compared to genes with 1 TF site (n=132, p<0.01). Most of the increase appears to be due to the fact that genes with multiple transcription factors are more likely to have a TATA box (p<0.01). Among genes without TATA boxes, genes with single transcription factor binding sites drifted at the same rate as genes with multiple transcription factor binding sites (p=0.94). Controlling for the number of TFs did not eliminate the increased expression divergence in genes with TATA boxes (p<0.01).

Wang et al. [6] measured the half life of S. *cerevisiae* mRNAs using a transcriptional shut-off assay. Core genes with TATA boxes had a mean half life of 32.5 minutes, which is significantly longer than genes without TATA boxes (mean=23.7, p<0.001). We found that genes with half lives longer than the median half life of 19 had a higher rate of divergence than those with half-lives shorter than 19 minutes (p<0.01). This relationship was confirmed in the Tirosh data (p=0.03), though there was no direct correlation between half-life of a gene's mRNA and its expression divergence. An increase in divergence rate was found in TATA-free genes, though the difference in expression divergence in genes containing TATA boxes did not reach statistical significance (p=0.18). Controlling for TATA status eliminated the effects of a long half-life in the Tirosh data.

7. Finding measurable gene duplication events

We created a set 41 genes from the non- *S. cerevisiae* yeasts that met the gene duplication criteria: the genes had two copies in one species only, both ORFs were complete, and, in order to ensure a duplication event and not a split ORF, both genes had to overlap the S. *cerevisiae* ortholog by >60%.

To ensure that these genes were unambiguously orthologous to the S. *cerevisiae* gene, we ran BLAST [7] searches of the three non- *S. cerevisiae* yeasts protein sequences against translated S. *cerevisiae* open reading frames using default parameters. We excluded 10 genes where at least one of the orthologs did not find the S. *cerevisiae* protein as the top hit.

Twenty-four of the remaining genes were measurably expressed in S. *cerevisiae* and the species with the duplication event. One gene was excluded because more than 10% of the reads aligning to the paralogs also aligned elsewhere in the genome. This left a set of 4, 5, and 14 genes in S. *paradoxus*, S. *mikatae*, and S. *bayanus* with probable lineage-specific duplication events with expression that could be measured using RNA-seq. Because S. *bayanus* had the most gene duplication events, we used this as our model.

8. Normalizing data

Read counts differ for each sample due to a variable number of reads produced by sequencing runs and the mixture of RNA within samples. Sample normalization is confounded by differences in gene expression. For example, if samples A and B are identical except that B contains one gene with

dramatically increased expression relative to A, a lower number of reads will be found for all other genes within sample B.

We therefore required a normalization factor to calculate expected values for our X^2 metric, and to calculate fold changes. We visualized the problem of normalizing read counts by plotting the read values on an X-Y plot and finding the line of best fit through the data. We used MATLAB to test various methods for finding the best slope. Robinson and Oshlack [8] present a solution to the problem of normalizing sample size using Trimmed M Means. However, any trimmed method requires parameters specifying how much to trim, and the determination of those parameters is somewhat arbitrary. We therefore did not test any methods that required parameters.

We visualized the problem by plotting simulated raw read counts (See Supplemental Methods 4 for simulation procedure) on a scatter plot with the counts for the genes in the control sample on the X axis and the test values on the Y axis. We then found the slope of the line that best fit the data points (Supplemental Figure S10). The assumption behind this model is that most genes will not be DE between samples. Genes should form a central cluster in the plot and the slope of the line going through the center of this cluster will indicate the scaling due to unequal sample sizes.

We used simulation data to test several methods of calculating this slope, simulating different experimental conditions, including unequal sample size and highly expressed outliers. The methods we tested were ordinary least squares regression, robust (weighted) regression, principal components analysis (PCA), normalization by total read count, and normalization by the median value of T_g/C_g. The technique that yielded the most accurate slope (m) when the true scaling factor was known was to take the median of ratio of test read counts to control read counts for each gene: $m = median\ (T_g/C_g)$. In particular, this method performed better than the frequently used approach of scaling data by total aligned read counts, which is not robust to highly expressed outliers (Supplemental Figure S10). Using total aligned reads could also bias results in cross-species studies where one genome will invariably be of higher quality and have fewer missing regions than the other.

The median method performed the best of the methods we tested, requires no parameters, and is symmetrical as to which sample is considered the control and test sample. It also has an advantage in that it is easily interpreted. Plotting the normalization lines through the data demonstrate that our normalization factors are reasonable (Figure 1 and Supplemental Figure S8).

Note that this method assumes that the datasets that are being normalized were prepared using the same library preparation protocol, as was the case in our experiment.

9. Measurements taken on the same day reproduced better

To assess the reproducibility of the RNA-Seq FC calls and to evaluate the validity of the confidence intervals, we compared the reproducibility of cross-species gene expression comparisons when the results were based on measurements taken when the samples from both species were prepared on the same day, versus measurements taken when they were prepared on different days. Samples labeled

replicate 1 for each of the four species were prepared on a different day than samples labeled replicate 2.

We counted the fraction of times where the second measurement value was within the confidence bounds given for the first measurement. Sixty-eight percent of genes would be expected to be within the 1 σ confidence interval and 95% at the 2 σ level. Cross-species FC calls made in samples prepared on the same day were reproduced close to the level predicted by our confidence intervals, with 52% and 86% of genes reproducing at the 1 and 2 σ levels (Supplemental Table S8). However, when comparisons were made of biological replicates prepared on different days (e.g. *S. cerevisiae* rep.1 vs. S. *paradoxus* rep. 2), only 36 and 73% of genes validated at the 1 and 2 σ levels. This discrepancy indicates that even subtle environmental differences in laboratory conditions on different days affect the consistency of the calls between species.

10. Calculation of fold change and the derivation of confidence interval around the log fold change

The fold-change was calculated as $T_{g(n)}/C_{g(n)}$ where $T_{g(n)}$ and $C_{g(n)}$ are the read counts for the test and control condition for a given gene, normalized by sample size, for each gene in the test and control samples. Reads were normalized by the *median* (T_g/C_g) . We added 0.01 to measurements that equaled zero to avoid divide by zero errors.

We calculated log2 fold changes as the log 2 value of (T_g/C_g).

We calculated confidence intervals around the log fold change by error propagation. The general formula for propagation of errors around x is as follows [9]:

$$\Delta f = \pm\sqrt{\sum_{i=1}^{n}\left(\frac{\partial f}{\partial x_i}\right)^2 (\Delta x_i)^2}$$

The confidence intervals around the fold change can be determined by first calculating the log fold change:

$$\log 2(F_{T/C}) = \log 2\left(\frac{\left(\frac{T_g}{T_{Exp}}\right)}{\left(\frac{C_g}{C_{Exp}}\right)}\right)$$

$$= log2\left(\frac{T_g}{C_g} \times \frac{C_{Exp}}{T_{Exp}}\right)$$

$$= log2\left(\frac{T_g}{C_g} \times \frac{1}{slope}\right)$$

$$= log2(T_g) - log2(C_g) \text{ -log2(slope)}$$

Then the partial derivative of the log fold change is taken with respect to C_g and T_g. Variance in the slope is assumed to be negligible.

$$\frac{\partial F}{\partial C_g} = \frac{-1}{C_g \times \ln(2)}$$

$$\frac{\partial F}{\partial T_g} = \frac{1}{T_g \times \ln(2)}$$

The variance in C is calculated similarly to the variance in C_{Exp} in the X^2 test statistic:

$${\sigma_{C_g}}^2 = C_g + \left(C_g * \sigma_{nP}\right)^2$$

Where the non-Poisson variance σ_{nP} is calculate as it is for the X^2 test statistic. Note that this equation is similar to the denominator of the chi square calculation except that the measured value is used instead of the expected value. This difference is due to the fact that in the fold change confidence interval we are calculating how confident we are that the fold change measurement is correct, rather than how closely the measurement corresponds to the null model that the gene is expressed at the same level in the control and test sample.

Using the standard formula for error propagation, we then calculate the $\sigma_{F_{\frac{T}{C}}}$ as follows:

$$\sigma_{F_{\frac{T}{C}}} = \sqrt{\left(\frac{-\sigma_{C_g}}{C_g \times \ln(2)}\right)^2 + \left(\frac{\sigma_{T_g}}{T_g \times \ln(2)}\right)^2}$$

SUPPLEMENTAL FIGURES

Supplemental Figure S1: Technical versus biological replicates

S1A

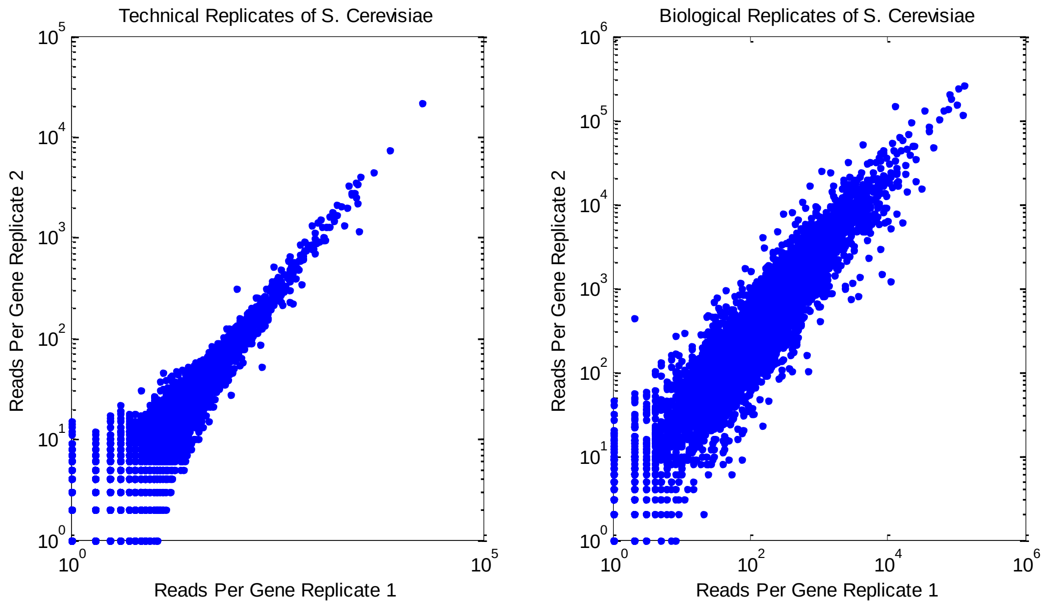

S1B.

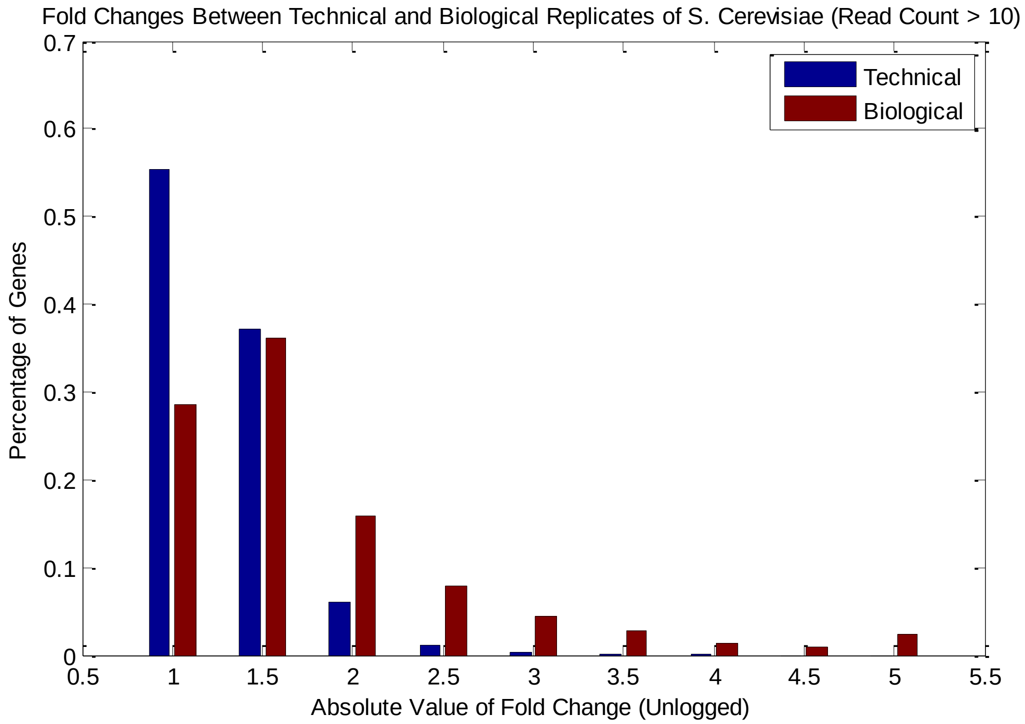

S1C

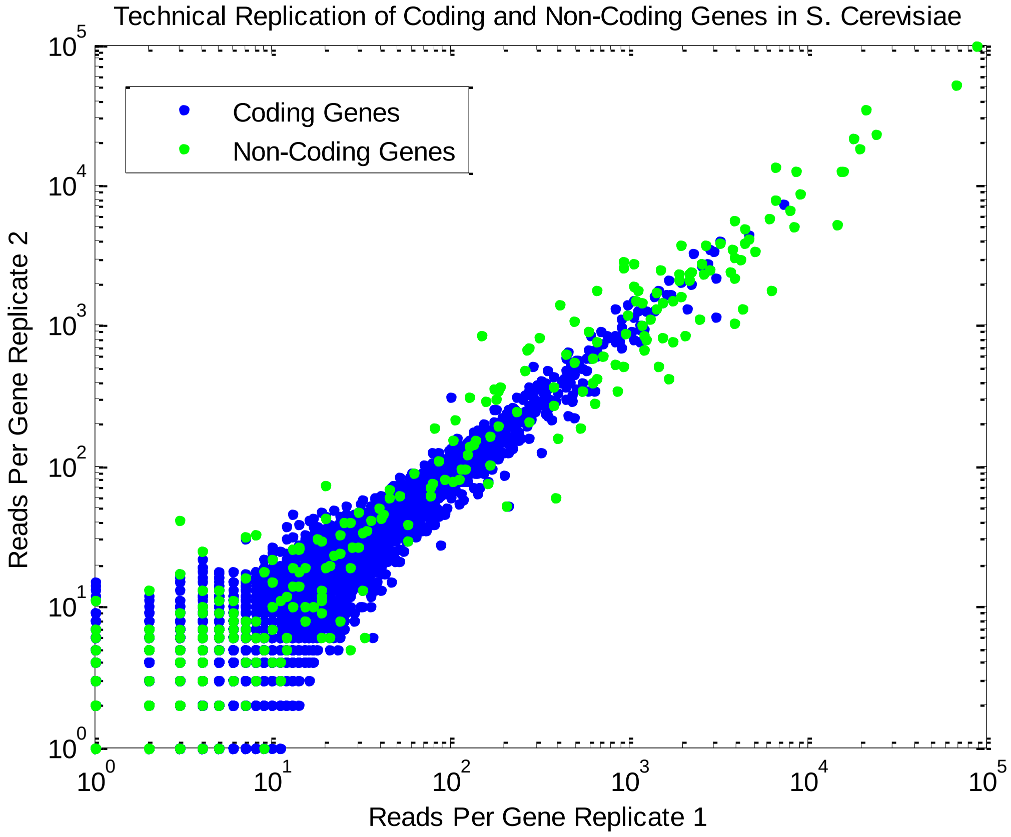

Supplemental Figure S1A: Replication of reads per coding gene in technical and biological replicates of S. S. cerevisiae S1B: The distribution of the number of coding genes which replicate with each fold change. For example, if a gene shows twice the number of reads (normalized for sample size) in replicate 1 than replicate 2 the fold change = 2X. Biological replicates show larger fold changes than technical replicates. S1C: Technical replication of coding genes versus non-coding genes. Non-coding genes primarily include snRNA, snoRNAs, and tRNAs.

Supplemental Figure S2

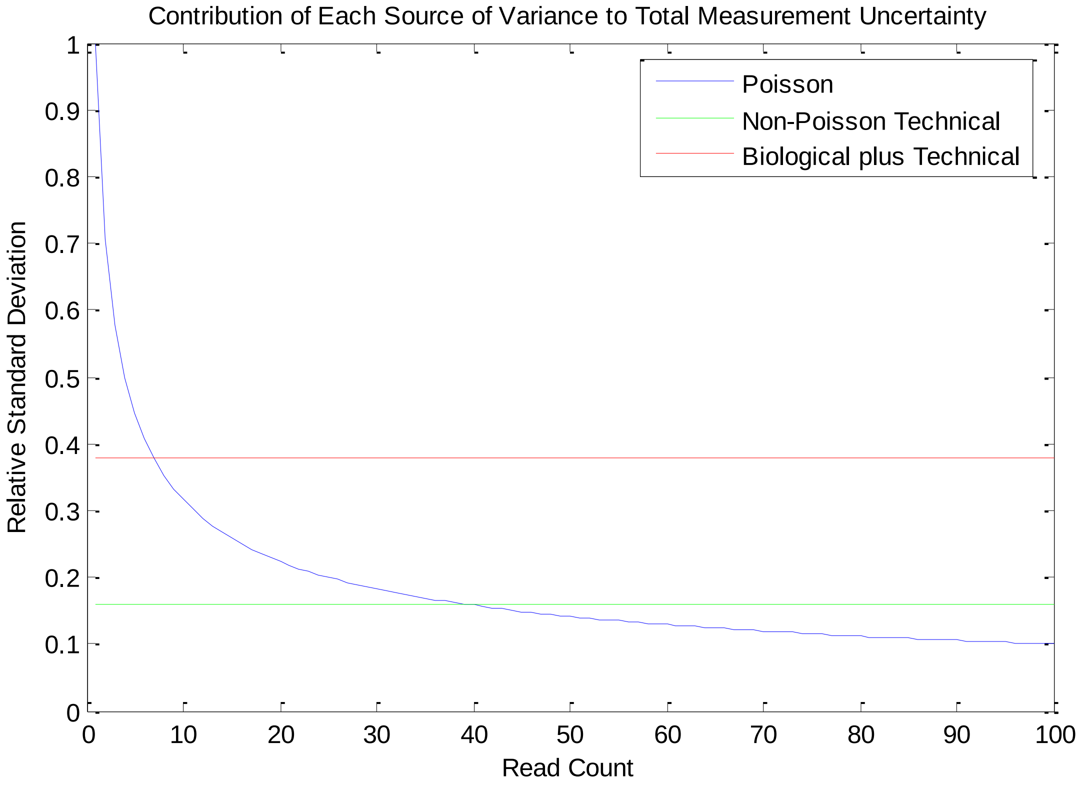

Supplemental Figure S2: The contribution of each source of variance to the total uncertainty in a gene's expression measurement at a given read depth. We used the relative standard deviation as the metric of uncertainty. The variance of a Poisson distribution for any count is equal to the count itself (R). The relative standard deviation due to Poisson counting noise is thus calculated as sqrt(R)/R. The Poisson error derives from on the count itself and is not experiment-specific. The amount of technical and biological variance shown is from the S. Cerevisiae technical and biological replicates. These values are the uniform over dispersion factor (U), whose calculation is described in the Methods section. The uniform value for the biological variance was not used in differential expression calculations, but represents a central value shown here for display purposes. The value of the technical replicate is 0.16 and the biological replicate is 0.38. We represent these as constants because we found that in our data the non-Poisson variance was uncorrelated with read depth. The uniform over dispersion is an aggregate value and the true relative standard deviation will be higher or lower than this value for individual genes. The over dispersion for the biological replicates includes both biological and non-Poisson technical variance. In interpreting this chart, note that uncorrelated variances (not the standard deviations) can be added to achieve the total variance.

Supplemental Figure S3

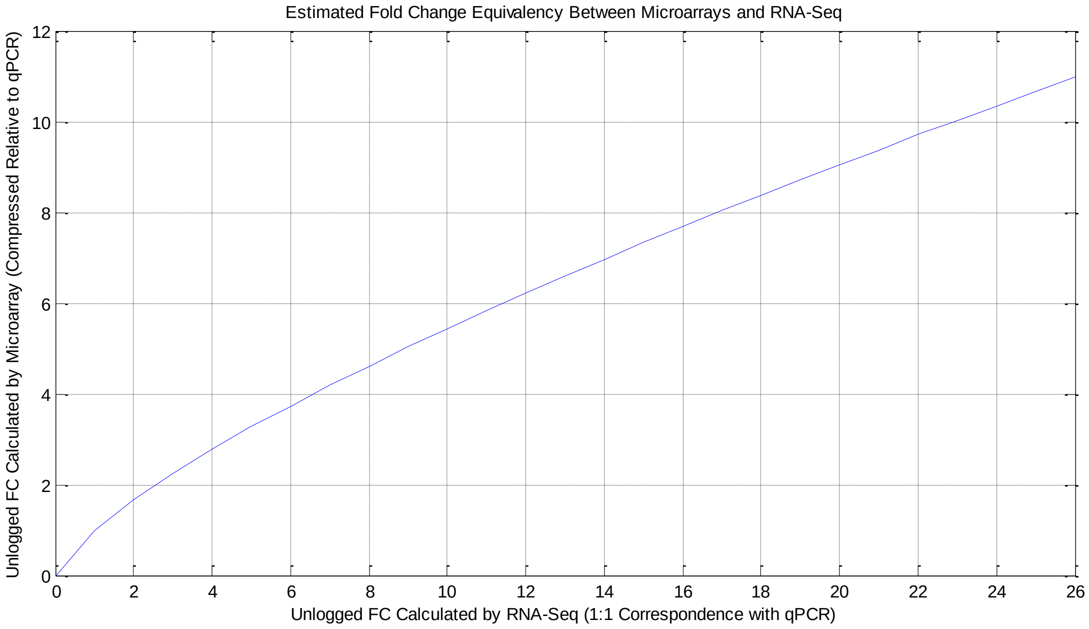

Supplemental Figure S3: Fold changes calculated by RNA-Seq are directly correlated with fold changes measured using qPCR. Microarray log2 fold changes are linearly correlated to log2 qPCR measurements with a slope of 1.23-1.49, depending on the method used to normalize the arrays [10]. Using the mean slope, fold changes measured by RNA-Seq at 2, 3, 4 are equivalent to a microarray FC (FCm) measured at 1.66, 2.24, and 2.77.

Supplementary Figure S4

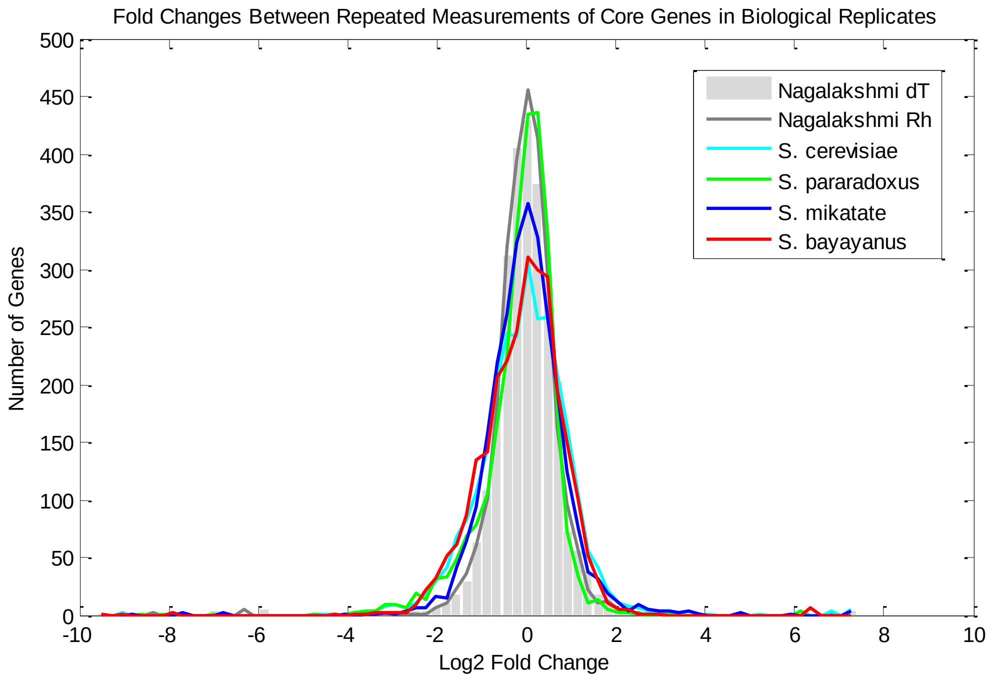

Supplemental Figure S4: Comparison of the fold changes that occurred between repeated measurements for the same gene in biological replicates in our replicates and in the Nagalakshmi dataset using our alignments pipeline, and the same set of core genes. We note that growth conditions were not exactly the same as the Nagalakshmi used an auxotrophic strain of yeast that was grown in YPAD rich media. Our strains were all prototrophic and grown in minimal media.

Supplemental Figure S5

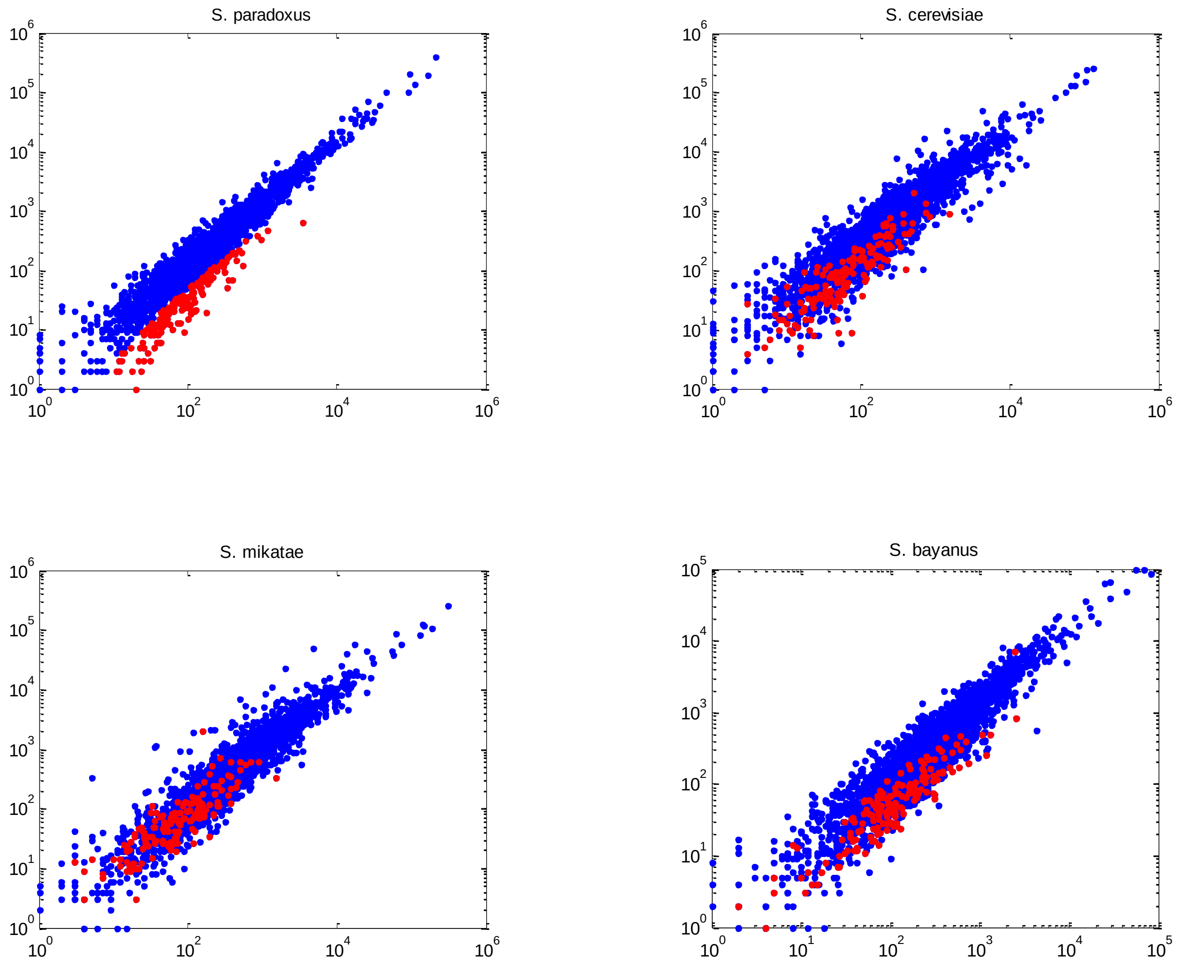

Supplemental Figure S5: We identified a set of 162 genes (red) that appeared to have higher expression in the second replicate of S. paradoxus versus the first. The orthologs of these genes are shown (red) to also be disproportionately unregulated in the second replicate of S. cerevisiae (90%), S. mikatae (62%), and S. bayanus (96%).

Supplemental Figure S6: Comparison of fold changes from RNA-seq versus qPCR

A. S. mikatae versus S. cerevisiae

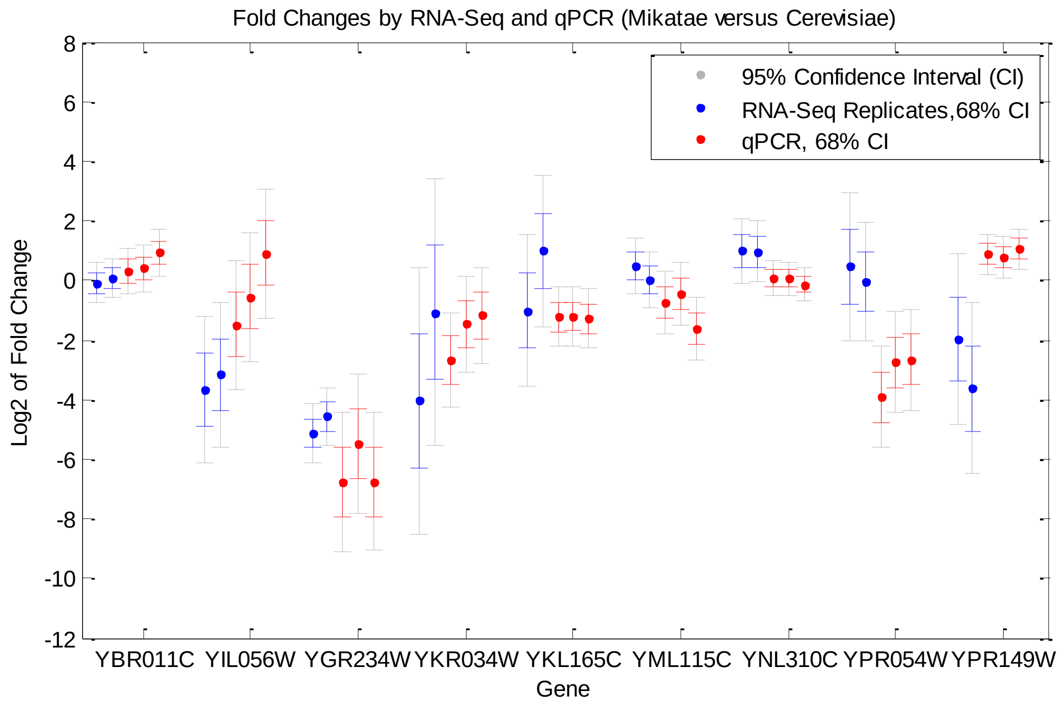

B. S. paradoxus versus S. cerevisiae

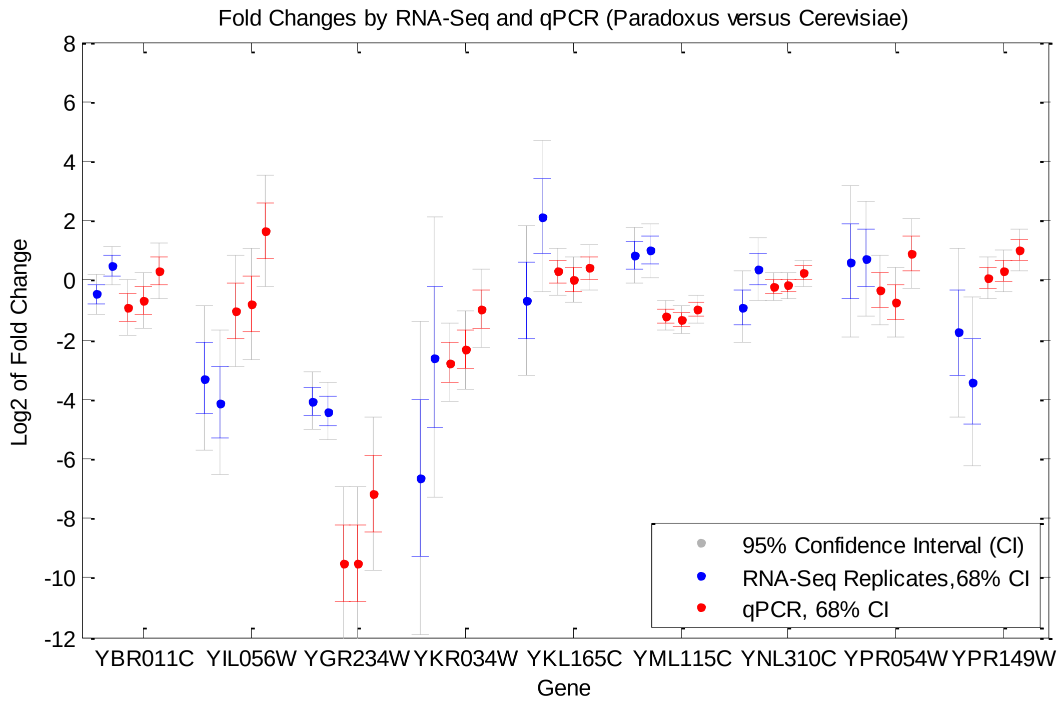

C. S. bayanus versus S. cerevisiae

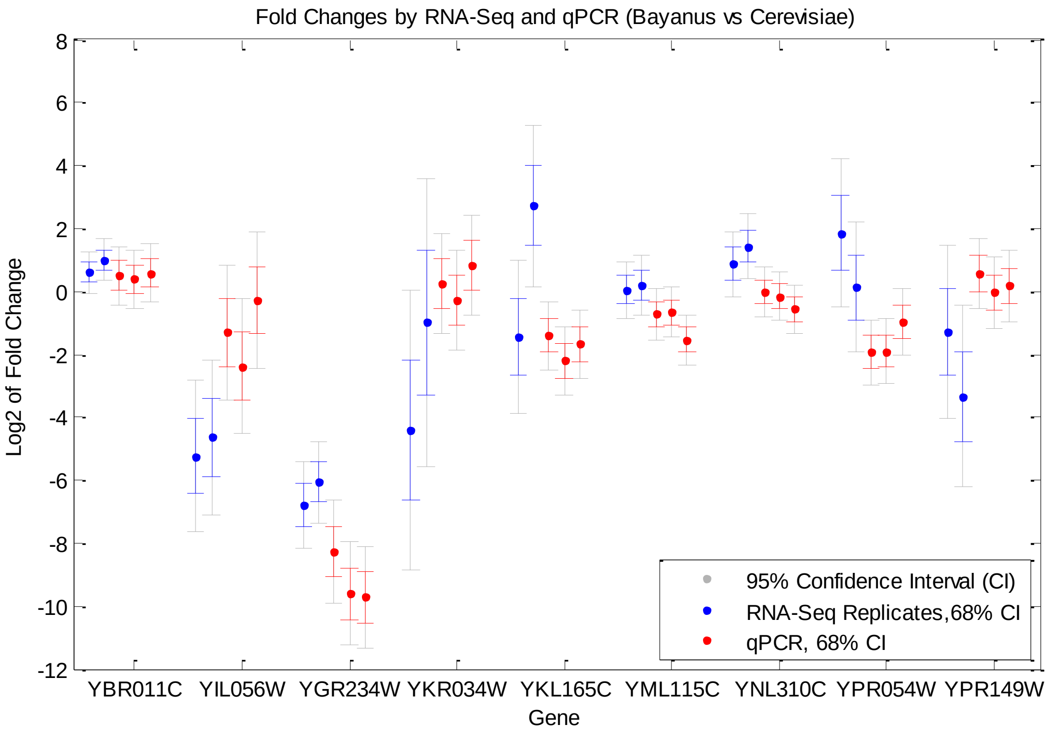

D. S. mikatae versus S. paradoxus

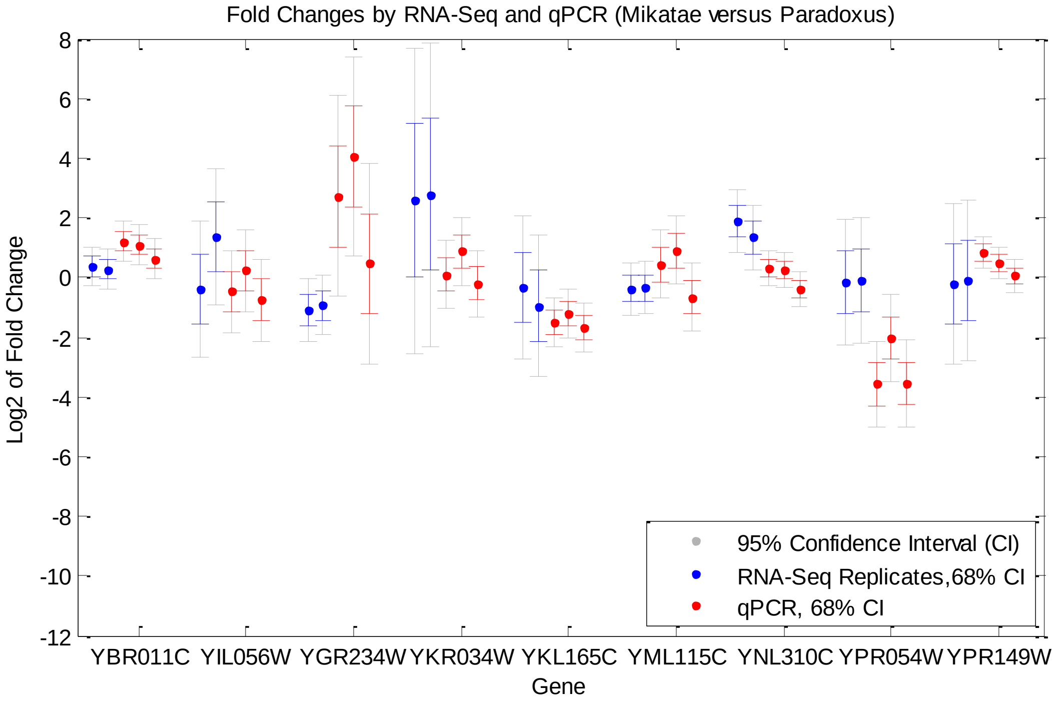

E. S. bayanus versus S. paradoxus

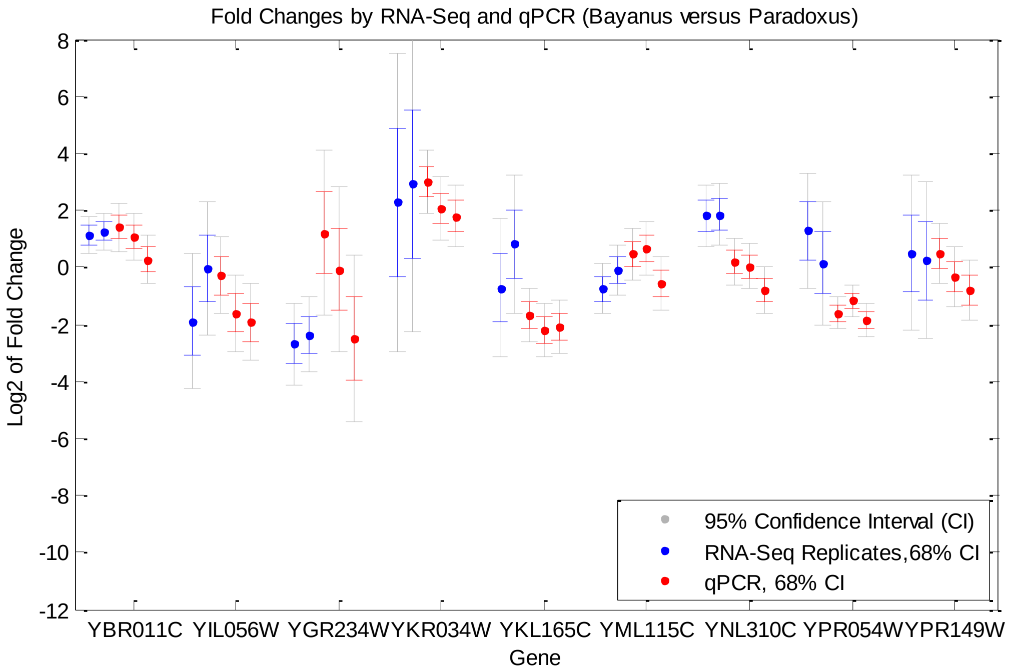

F. S. bayanus versus S. mikatae

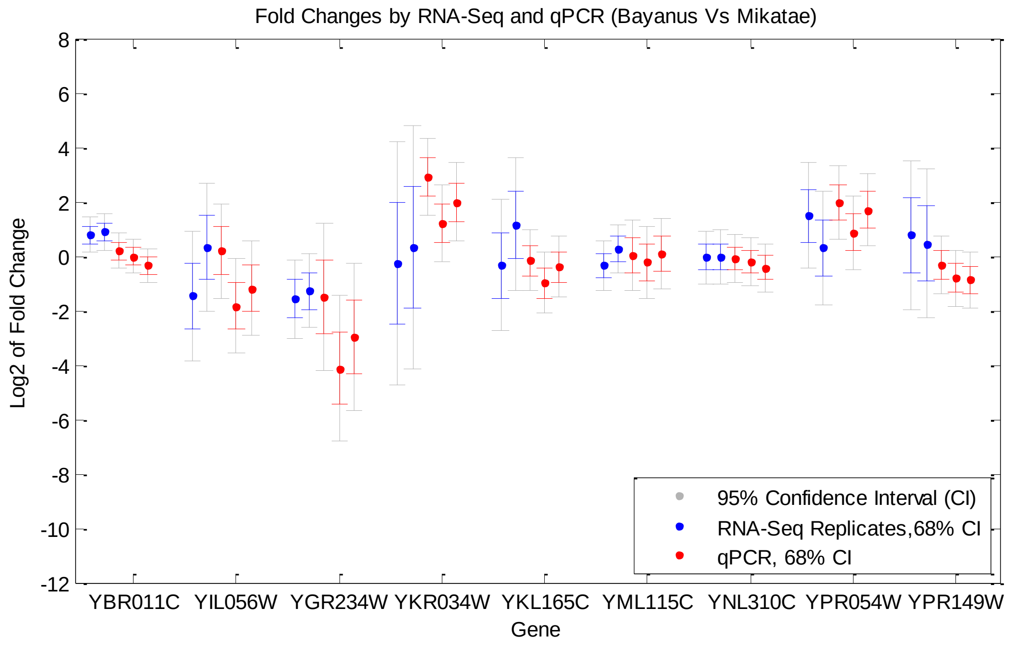

Supplemental Figure S6: Fold changes calculated by RNA-Seq and qPCR for the comparison. Each bar represents the comparison between a single biological replicate pair. Colored bars represent the 1 σ (68%) confidence intervals around fold-change calculations. Gray bars represent 2 σ (95%) confidence intervals.

Supplemental Figure S7A

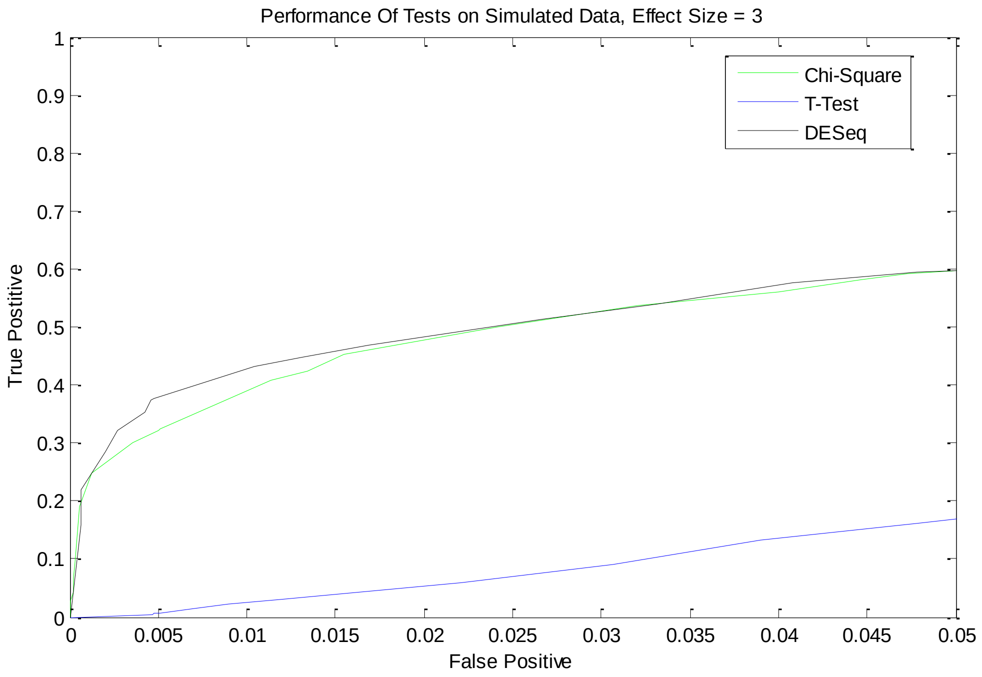

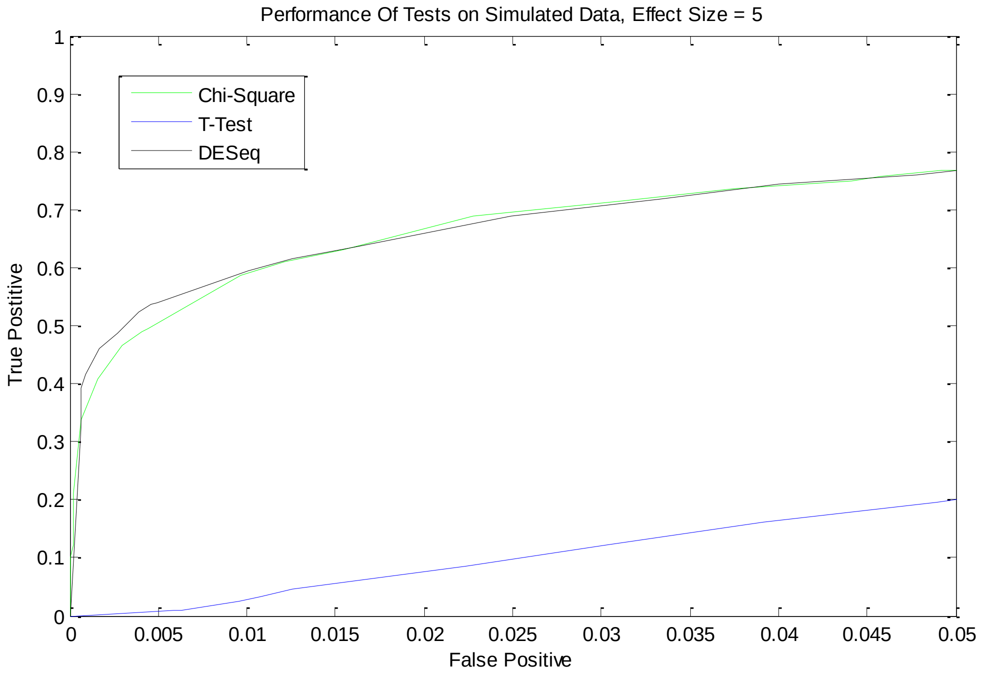

Supplemental Figure S7A: Comparison of performance of statistical methods. Differential expression is modeled as a random change in expression of the effect size magnitude specified.

Supplemental Figure S7B

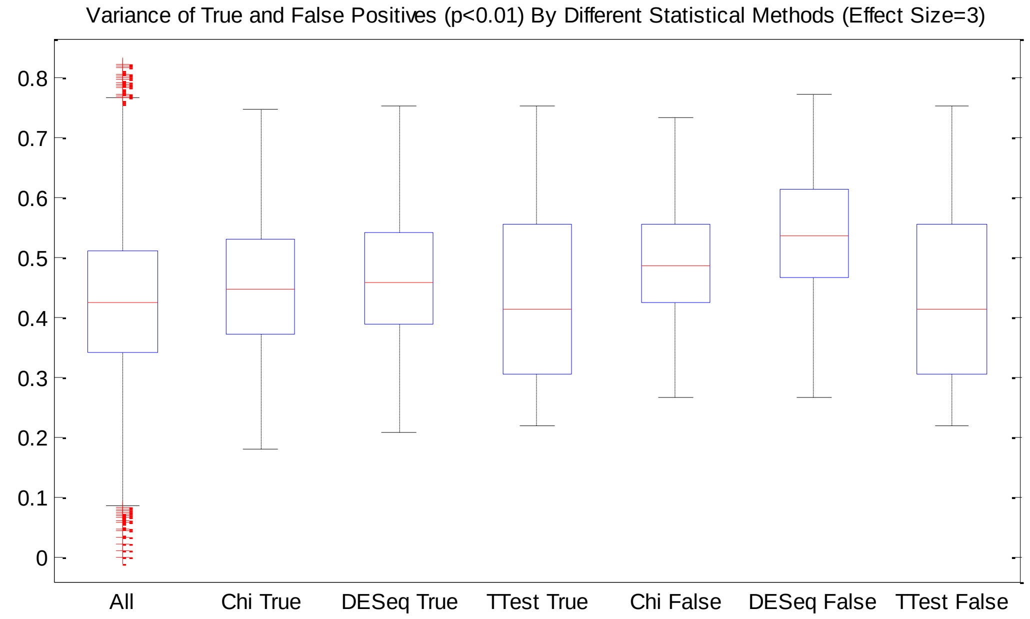

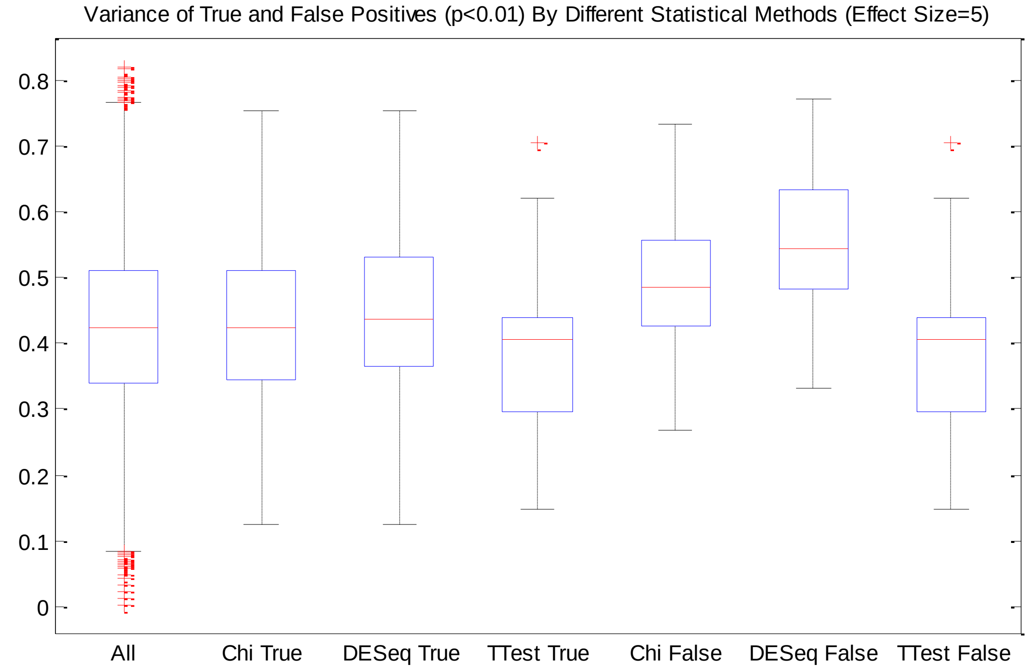

Supplemental Figure S7B: The variance of genes called true and false positives by the statistical methods. The metric we use for variance here is the gene-specific relative standard deviation that was used in the data simulation. This number includes both technical and biological variability. True and false positives were modeled randomly without respect to variance (See Supplemental Methods 4).

Supplementary Figure S7C

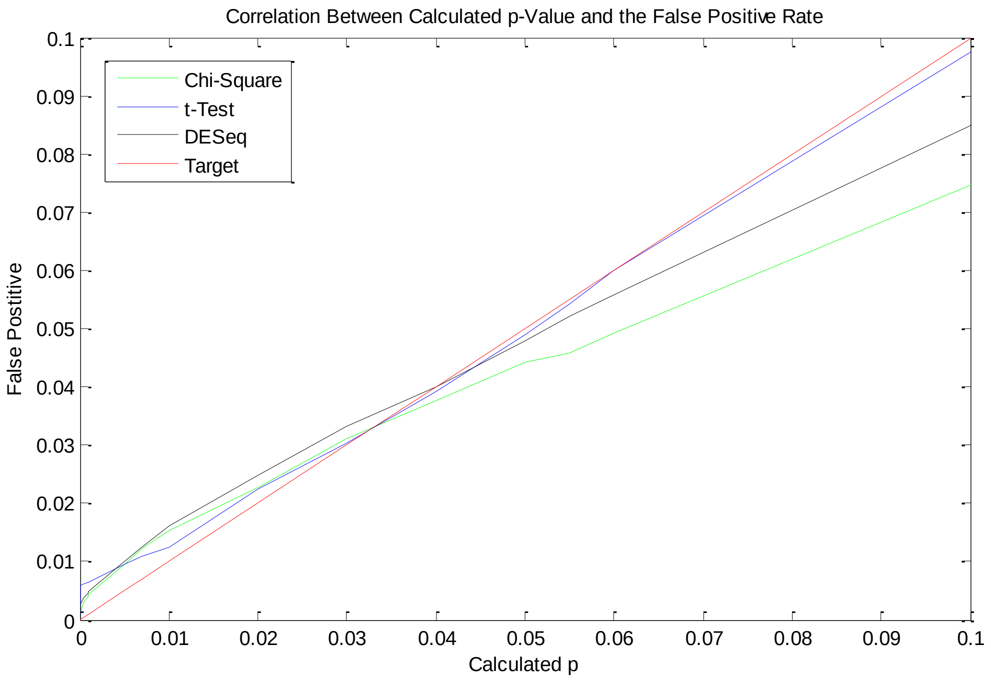

Supplementary Figure S7C: Correlation between the calculated p value and the false positive rate. The red line represents the target value

Supplemental Figure S7D

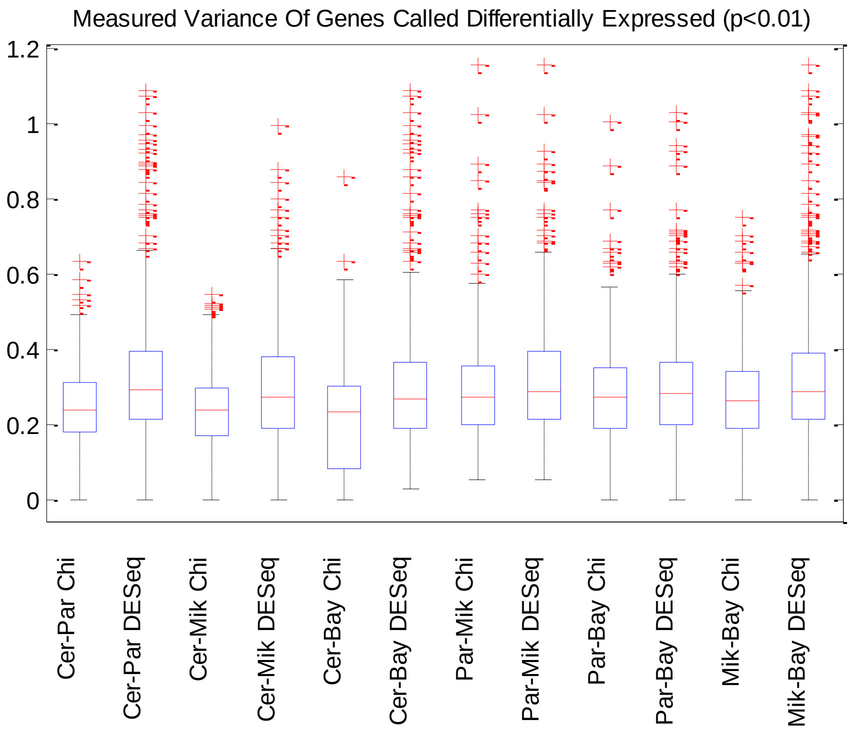

Supplemental Figure S7D: Comparison of the measured variance of genes called differentially expressed by DESeq versus our method. Red crosses represent outliers.

Supplemental Figure S8: Differential expression in pairwise comparisons

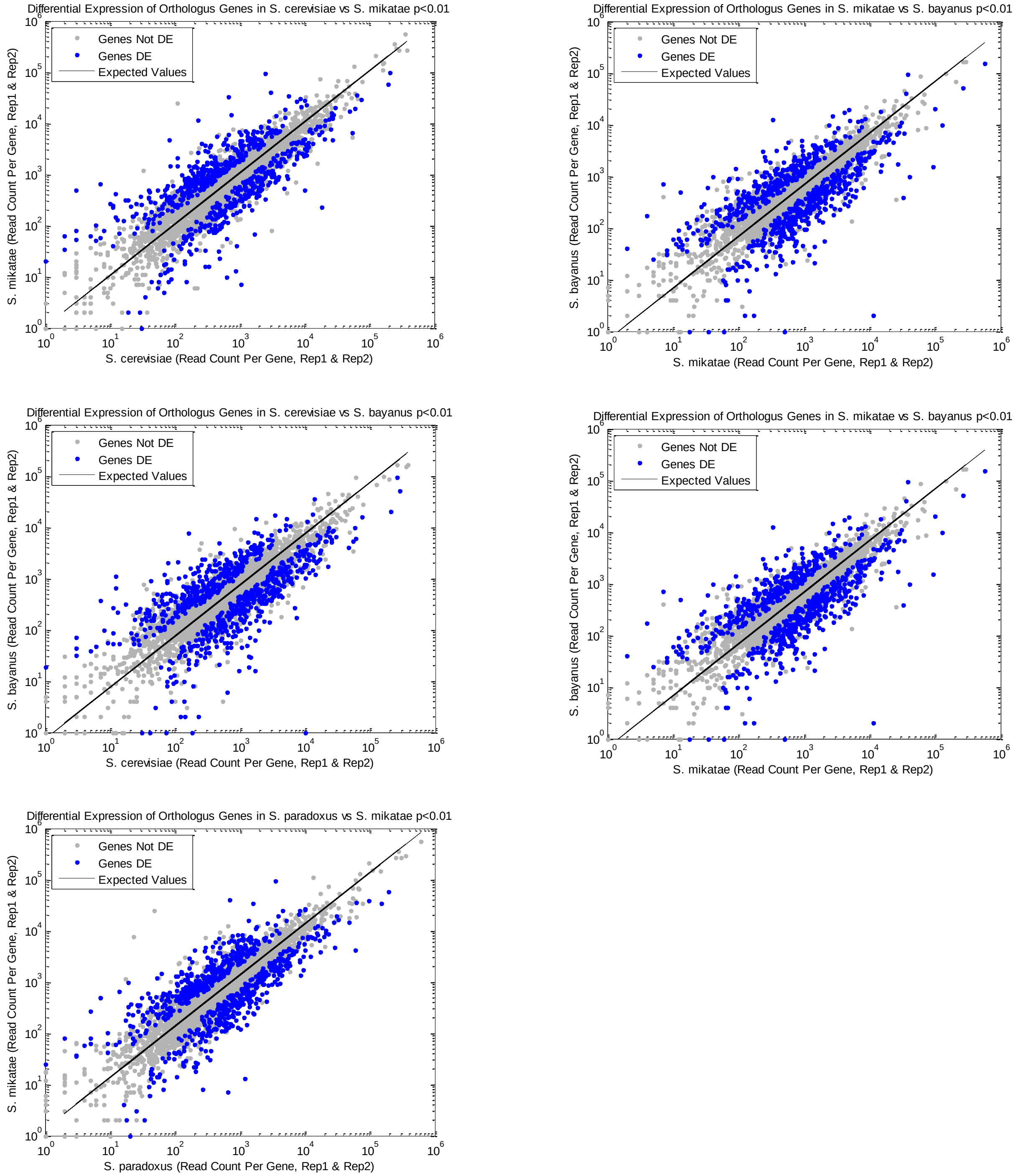

Supplemental Figure S8: Differential gene expression in cross-species pairwise comparisons. Plotted values are the number of reads uniquely aligning to the gene. Differentially expressed genes (blue) are measured at $p<0.01$.

Supplemental Figure S9A:

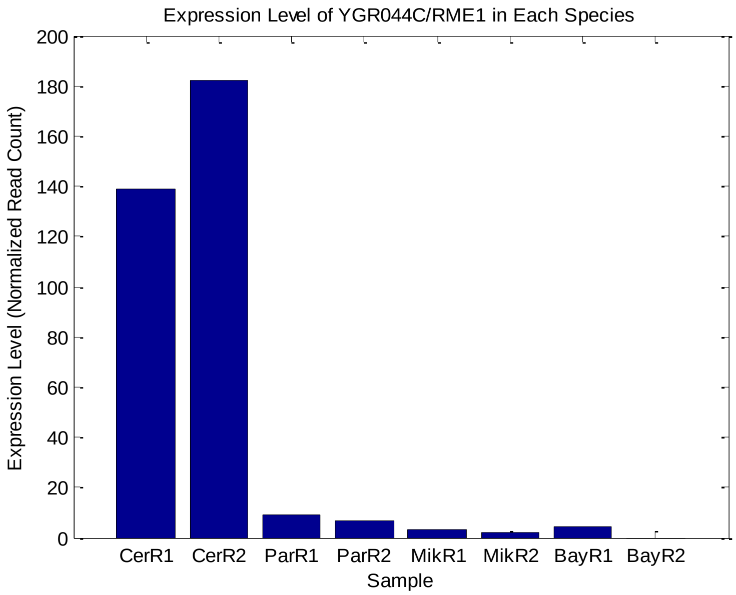

Supplementary Figure S9B:

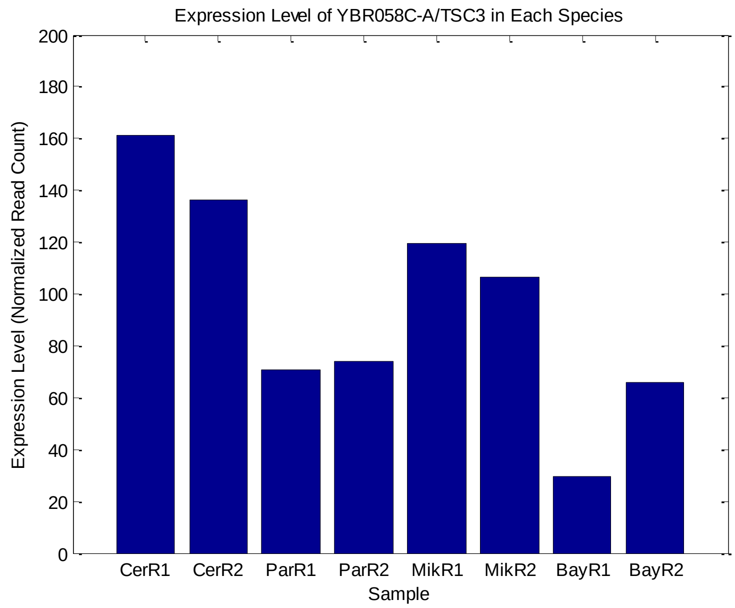

Supplementary Figure S9: A) Lineage-specific increase of expression in S. *cerevisiae* versus the three other species for the gene RME1. B) Increase of expression in S. *cerevisiae* versus S. bayanus, but intermediate expression in S. mikatae for the gene TSC3.

Supplemental Figure S10A:

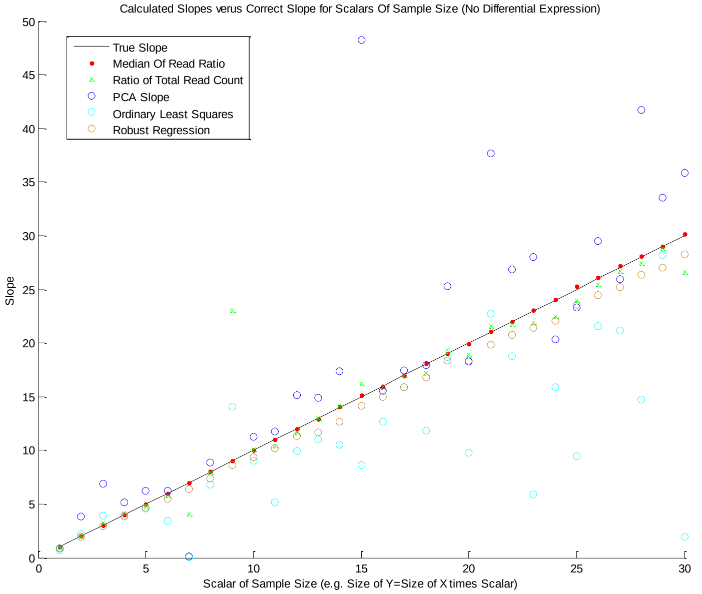

Supplemental Figure S10B:

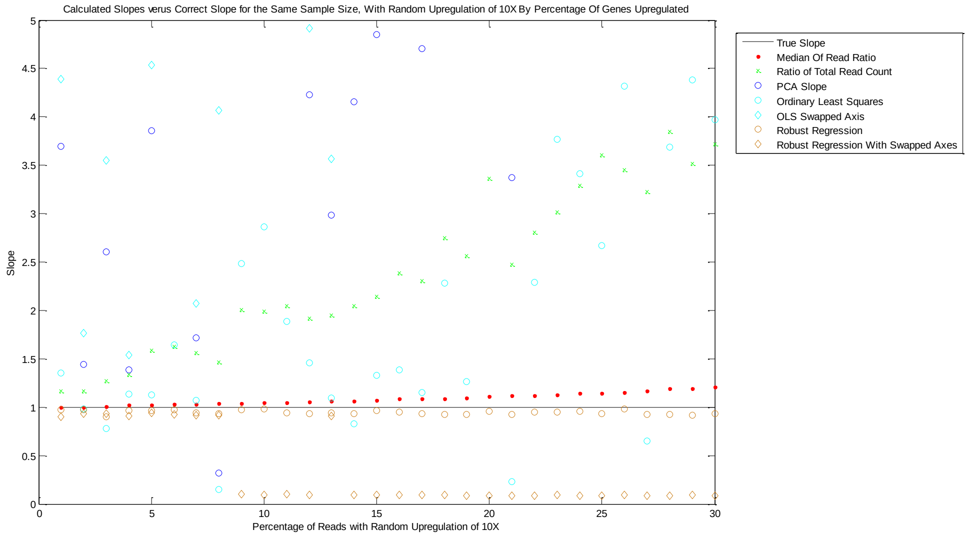

Supplemental Figure S10: Performance of calculated versus actual slopes using different methods for finding the correct slope. Methods tested were the median of the ratio of reads, the ratio of the total read counts in both samples, principle components analysis (PCA), and ordinary least squares (OLS) and robust regression. Figure 10A shows the performance of these methods when the sample sizes vary between conditions. Figure 10B shows the effects of adding a group of DE genes to one of the samples. Robust and OLS regression are sensitive to which samples are chosen as the control (X axis) and test (Y axis). Regression results are presented twice in 10B with the X and Y axes swapped.

Supplemental Figure S11

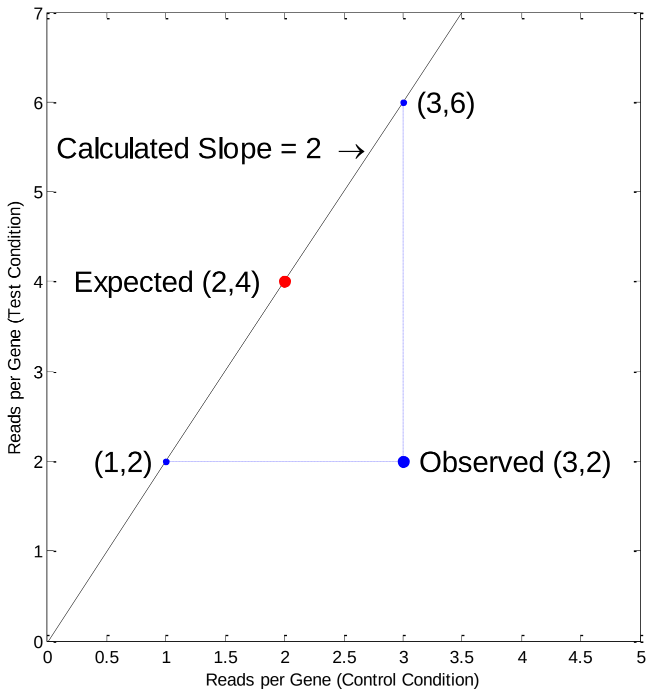

Supplemental Figure S11: Calculation of expected values (means) in our chi square. The expected value is found by taking the midpoint of the segment of the slope line that is bounded by the points (C_g, $C_g m$) and (T_g/m, T_g), where C_g is the reads per gene in the control condition, T_g is the reads per gene in the test condition, and m is the slope of the line that normalizes the two samples. In this example, $C_g=3$, $T_g=2$, and m=2. The boundaries of the line segment are thus (3, 6) and (1,2). The midpoint of this line segment (4,2) gives unbiased weighting to both samples. The orthogonal point, by contrast, gives more weighting to the more highly measured sample.

Supplementary Figure S12:

A

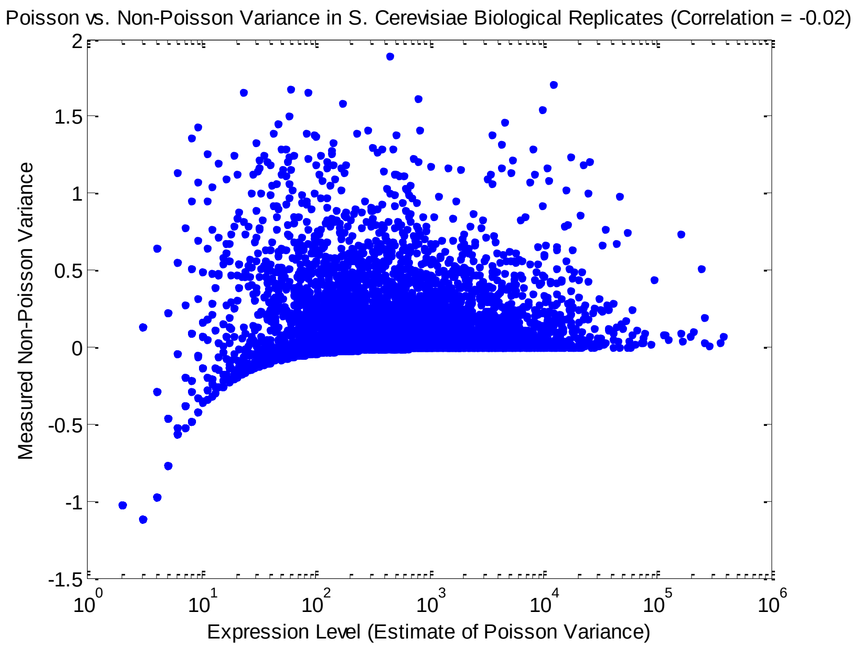

B

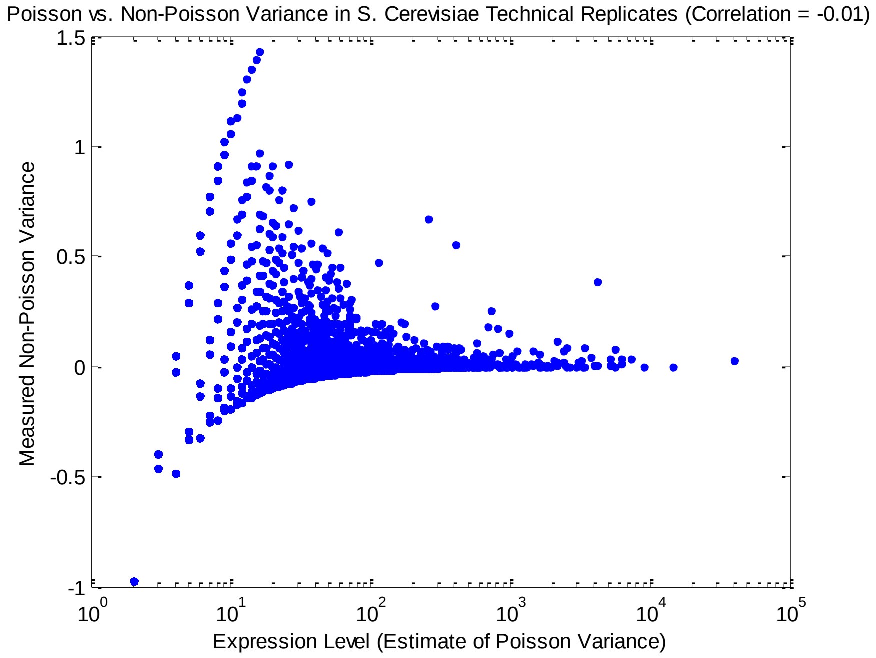

Supplemental Figure S12: Estimated Poisson and non-Poisson variance are uncorrelated in biological (A) and technical (B) replicates. Data points represent coding genes measured in both replicates. Calculation of relative non-Poisson variance is as described in the Methods section.

Supplementary Figure S13:

S13 A

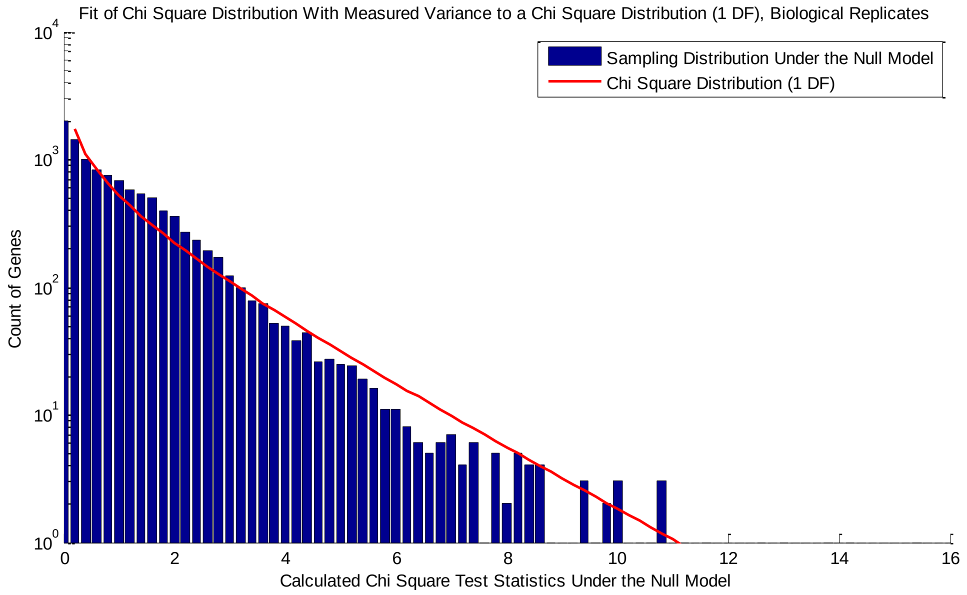

S13 B

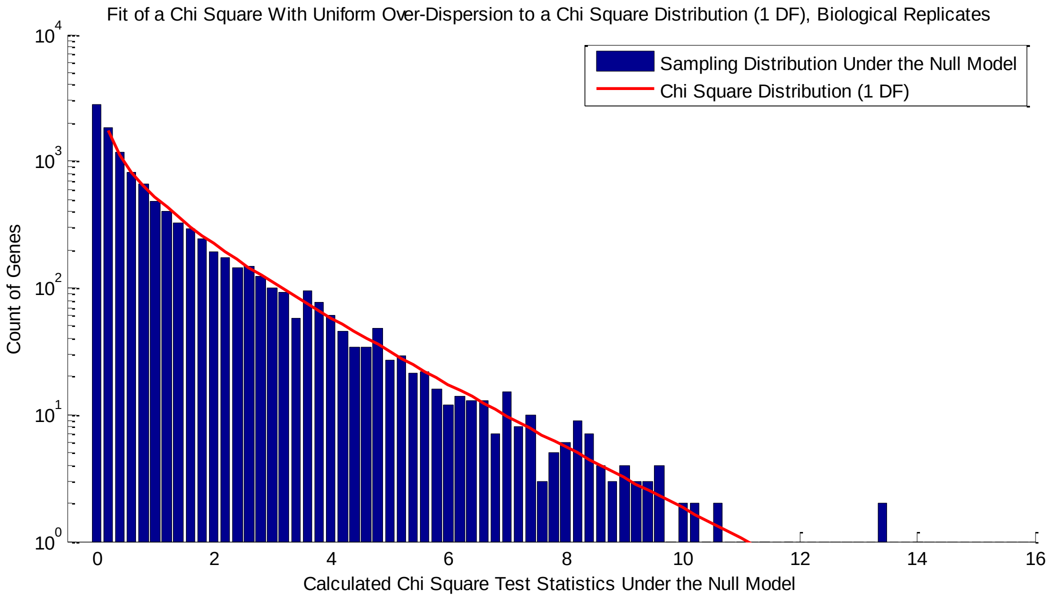

S13 C

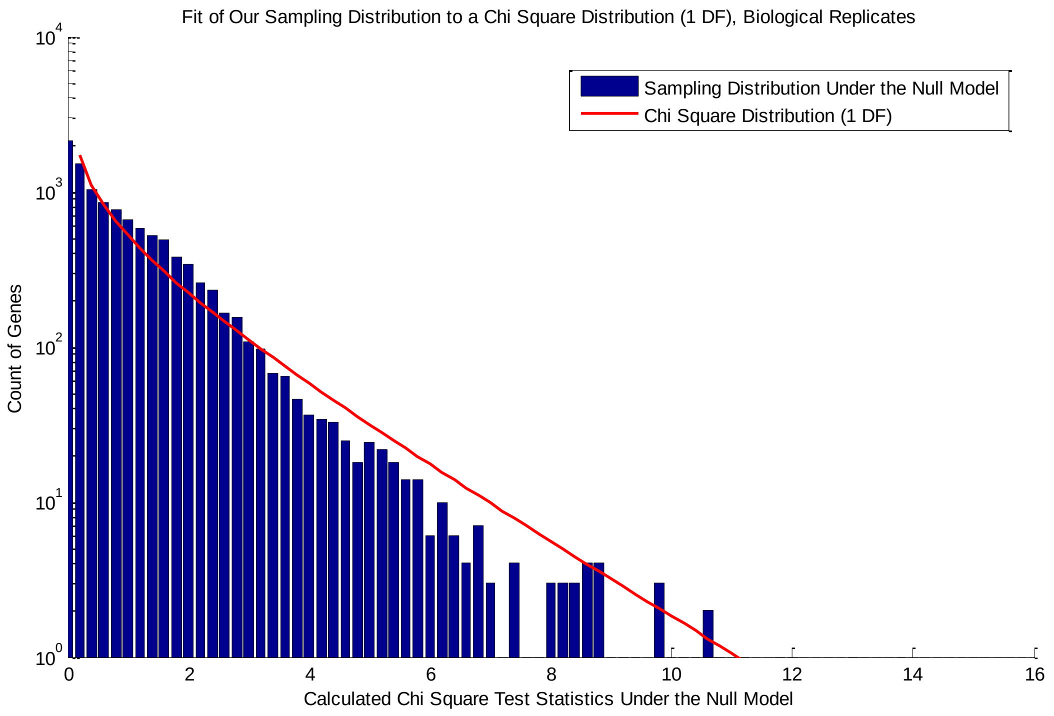

Supplemental Figure S13: When the assumptions of a X^2 test with two conditions are true, a sample of X^2 test statistics calculated under a null model will be well approximated by a X^2 distribution with one degree of freedom (DF). We show that the when we apply our X^2 test to biological replicates (the null model), we generate a sampling distribution that is that is well modeled with measured variance (A) or uniform over-dispersion (B) is used. Our final X^2 test is shown in Figure C. The replicate data from all four species was used.

Supplemental Figure S14: Fit of parameters to mathematical distributions

S14 A.

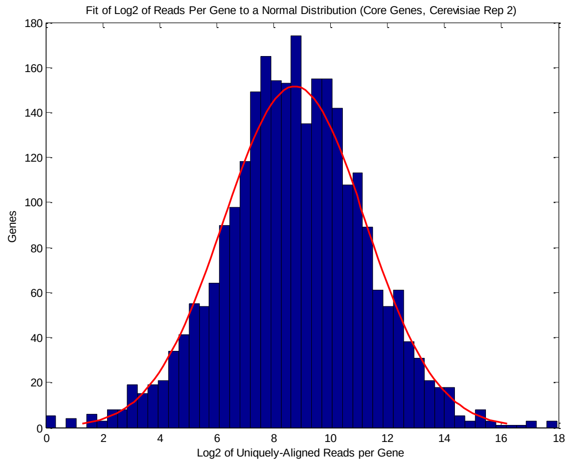

Supplemental Figure14A: Log2 of uniquely aligned read counts per gene fit to a normal distribution. Genes with 0 reads were excluded (n=5). The fit of the log of the values to a normal distribution shows that the underlying distribution is lognormal, and the fit is easier to visualize than the unlogged values fit to a lognormal distribution.

S14 B

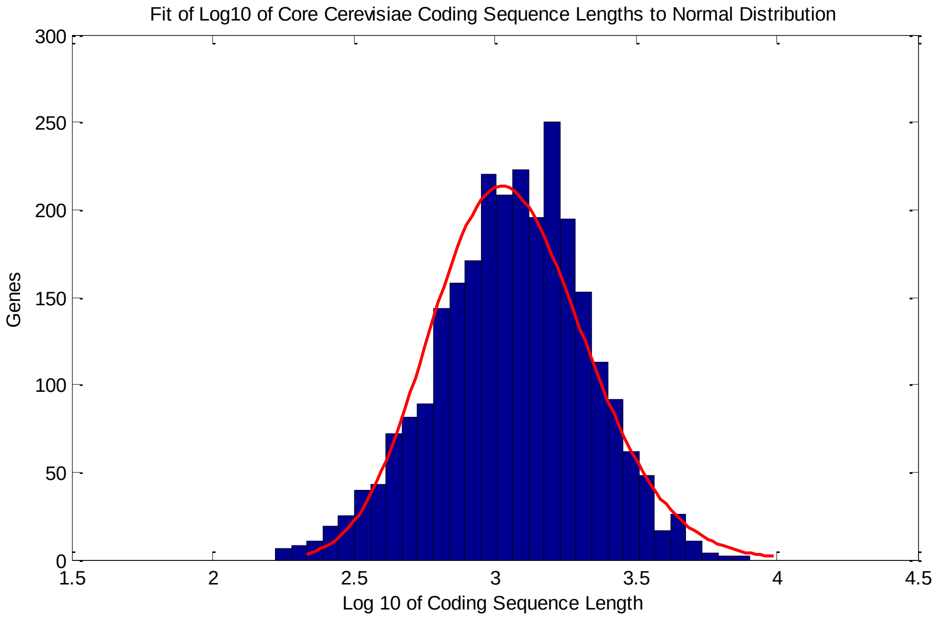

Supplemental Figure S14B: The log of the length of CDS's from conserved single-exon S. *cerevisiae* genes fit to a normal distribution. Non-conserved genes do not fit the distribution as well (not shown).

Supplemental Figure S15A: Simulated Biological Replicates

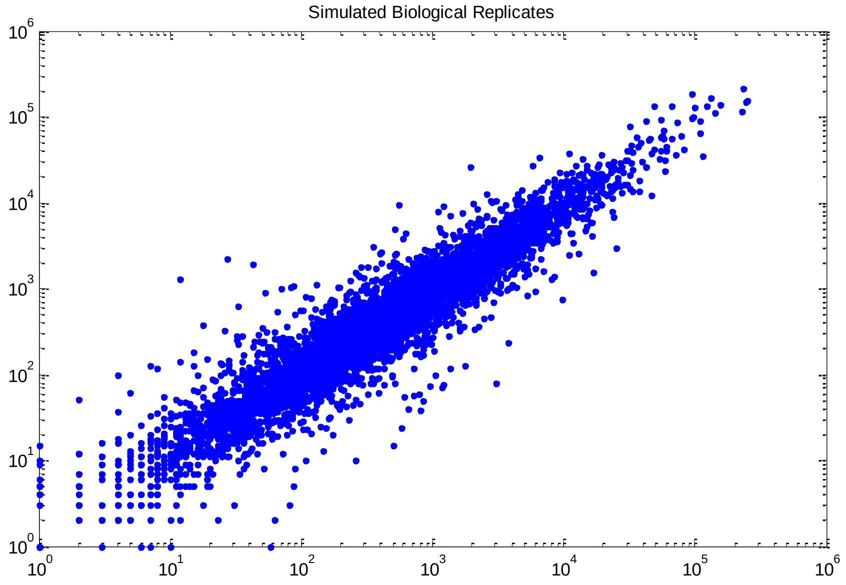

Supplemental Figure 15B: Simulated Variance

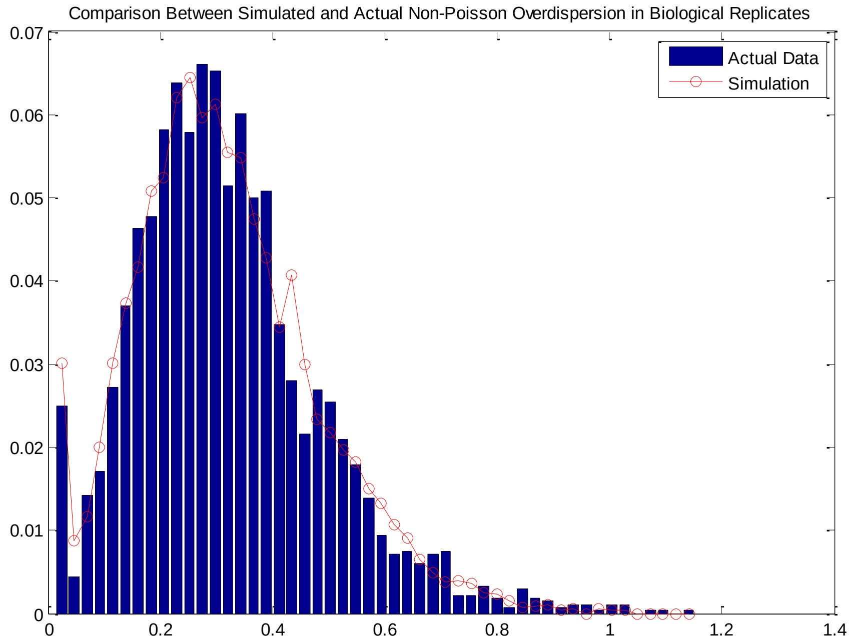

Supplemental Figure S15: Results from simulation procedure to generate biological replicates with a gene expression level and variance modeled on actual data. A) Simulated biological replicates. B) Correspondence between total non-Poisson overdispersion (measured relative standard deviation, σ_{nP}) in real versus simulated samples.

Supplemental Tables

Supplemental Table S1: Alignment Results

**Species** (Coverage of Reference)		**Reads**	**Reads Aligned**		**Reads Aligned Unique**		**Reads 95% Correct**	**Reads 95% Correct Aligned**	
S. cerevisiae (Finished)	Rep1	34,760,166	19,311,072	56%	7,254,726	21%	25,090	23,998	96%
	Rep2	48,982,408	27,681,246	57%	16,891,689	34%	39,759	38,913	98%
S. paradoxus (7.7 X)	Rep1	31,460,846	18,498,038	59%	10,186,790	32%	29,372	27,988	95%
	Rep2	38,286,247	23,076,712	60%	13,112,605	34%	39,469	38,145	97%
S. mikatae (5.9 X)	Rep1	39,345,872	20,294,230	52%	18,128,566	46%	28,289	26,068	92%
	Rep2	38,716,464	21,350,790	55%	19,090,152	49%	35,165	33,025	94%
S. bayanus (6.4 X)	Rep1	27,834,835	14,895,197	54%	12,840,218	46%	17,259	16,571	96%
	Rep2	32,964,873	19,052,389	58%	15,731,140	48%	28,321	26,688	94%
**Total**		**292,351,707**	**164,293,232**	**56%**	**113,361,995**	**39%**	**242,724**	**231,396**	**95%**

Supplemental Table S1: Reads 95% Correct are those reads where the quality scores indicate that the read has a 95% probability of being completely accurate. To obtain this, the quality score for each transition call (analogous to a base call in other sequencing technologies) is backed into the percentage probability that the call is correct. These individual probabilities are then multiplied serially to get the probability that all transition calls in the read are correct. These will represent the highest quality reads in the dataset. Total alignment rates of these high quality reads were: S. *cerevisiae* 97%, *S. paradoxus* 96%, S. *mikatae* 93%, and S. *bayanus* 95%.

Supplemental Table S2: Fold changes observed for the same gene between measurements in biological versus technical replicates

**Replicate Type**	**Measured Genes**	**Pearson Correlation**	**Mean Absolute FC**	**FC at 95th Percentile**	**FC at 99th Percentile**	**Maximum FC**
Technical	2343	97.5	1.35	2.1	3.0	6.5
Biological	4751	90.8	1.84	3.6	6.9	25

Fold changes are the postive fold change for each gene between species. (Higher Expression/Lower Expression, normalized read counts.) To reduce the effects of Poisson counting noise as a source of variance, genes measured with fewer than 10 reads in either sample were excluded.

Supplemental Table S3: qPCR measurements

	S. cerevisiae			S. paradoxus			S. mikatae			S. bayanus		
YKR034W	0.009	0.007	0.003	0.010	0.006	0.006	0.001	0.003	0.002	0.001	0.001	0.002
YGR234W	0.151	0.211	0.144	0.001	0.000	0.000	0.001	0.005	0.001	0.000	0.000	0.001
YIL056W	0.272	0.315	0.065	0.111	0.061	0.053	0.097	0.215	0.121	0.135	0.183	0.204
YPR149W	2.921	2.885	2.009	4.319	2.824	2.271	5.395	4.957	4.170	3.034	3.556	4.013
YPR054W	0.007	0.007	0.003	0.002	0.002	0.002	0.001	0.001	0.001	0.006	0.004	0.006
YNL310C	0.008	0.009	0.007	0.007	0.008	0.005	0.008	0.009	0.006	0.007	0.008	0.008
YML115C	0.023	0.023	0.024	0.014	0.015	0.008	0.014	0.017	0.008	0.010	0.009	0.013
YKL165C	0.472	0.541	0.336	0.179	0.118	0.105	0.201	0.234	0.139	0.574	0.549	0.446
YBR011C	2.953	3.281	1.969	4.183	4.312	2.938	3.653	4.333	3.742	1.583	2.044	2.424

Supplementary Table S3: qPCR measurements for three additional biological replicates of each species.

Supplemental Table S4: Comparison of genes called differentially expressed by methods

	Chi	DESeq	Both	% of All Called Genes That Were Called By Both Methods
S. cerevisiae- - S. paradoxus	344	497	294	54%
S. cerevisiae- S. mikatae	366	424	270	52%
S. cerevisiae- S. bayanus	439	484	309	50%
S. paradoxus- S. mikatae	458	645	426	63%
S. paradoxus- S. bayanus	494	666	448	63%
S. mikatae- S. bayanus	478	601	407	61%
Total	2579	3317	2154	58%

Supplemental Table S4: The count of genes called differentially expressed at p<0.01 between methods.

Supplemental Table S5A: Genes Differentially Expressed in Each Branch of the Phylogeny (p<0.01)

S. cerevisiae	Up	YAL037W, YBR004C, YBR127C, YBR157C, YBR283C, YCL011C, YCL021W-A, YDL046W, YDL048C, YDL124W, YDL237W, YDR265W, YDR284C, YDR319C, YDR378C, YDR497C, YDR533C, YEL002C, YEL025C, YER021W, YFL029C, YGL245W, YGR026W, YGR044C, YGR117C, YGR234W, YHL019C, YHR002W, YHR063C, YHR143W, YIL023C, YIL050W, YIL088C, YIL119C, YIR030C, YIR031C, YJL035C, YJL153C, YJR011C, YJR073C, YKL013C, YKL038W, YKL210W, YKL211C, YKR030W, YLL038C, YLR325C, YML101C, YMR180C, YMR216C, YMR291W, YNL090W, YNR036C, YOL108C, YOR089C, YOR099W, YOR103C, YOR176W, YOR212W, YOR220W, YOR261C, YOR348C, YPL068C, YPR066W
	Down	YAL008W, YBL069W, YBR088C, YBR205W, YBR247C, YBR272C, YCL025C, YDL086W, YDL101C, YDL103C, YDR009W, YDR043C, YDR109C, YDR115W, YDR183W, YDR206W, YDR374C, YDR479C, YER038C, YER071C, YER101C, YFL003C, YFL038C, YGL091C, YGL121C, YGL128C, YGL241W, YGR102C, YGR199W, YGR277C, YHR075C, YHR122W, YHR127W, YHR143W-A, YHR160C, YHR202W, YHR208W, YIL033C, YJL003W, YJL029C, YJL058C, YJL157C, YJL184W, YJR013W, YJR069C, YKL103C, YKL107W, YKR038C, YKR076W, YLR042C, YML066C, YML070W, YMR055C, YMR095C, YMR099C, YMR154C, YMR200W, YMR300C, YNL191W, YNL219C, YNL316C, YOL080C, YOL104C, YOL137W, YOL139C, YOL154W, YPL103C, YPL123C, YPL214C, YPR151C
S. paradoxus	Up	YAL042W, YBR107C, YBR166C, YBR201W, YBR250W, YBR252W, YBR259W, YBR272C, YCR002C, YCR065W, YCR068W, YDL120W, YDL198C, YDR067C, YDR320C-A, YDR368W, YDR374C, YDR519W, YDR539W, YEL001C, YER157W, YER174C, YER183C, YGL094C, YGL098W, YGL123W, YGL158W, YGL220W, YGL240W, YGL255W, YGL257C, YGR012W, YGR014W, YHR005C, YHR129C, YHR187W, YIL043C, YJR017C, YJR149W, YKL024C, YKL068W, YKL084W, YKL154W, YKR091W, YLL049W, YLR010C, YLR042C, YML112W, YMR152W, YNL010W, YNL217W, YNL316C, YOL054W, YOL086W-A, YOL097C, YOR166C, YOR221C, YOR289W, YOR297C, YOR381W, YPL144W, YPL252C, YPR113W, YPR147C, YPR174C
	Down	YAL009W, YAL060W, YBR052C, YBR126C, YBR127C, YBR146W, YBR254C, YBR286W, YCL005W, YCR054C, YDL008W, YDL119C, YDL181W, YDL199C, YDR054C, YDR078C, YDR322C-A, YDR429C, YER145C, YFL017W-A, YFL034W, YFL041W, YFR048W, YFR049W, YGR075C, YGR250C, YHR009C, YHR028C, YHR143W, YIL050W, YIL051C, YIL064W, YIL119C, YJR085C, YJR144W, YKL085W, YKL137W, YKL176C, YKR049C, YKR050W, YKR085C, YLR215C, YLR231C, YLR327C, YML120C, YMR196W, YMR238W, YOL151W, YOR006C, YOR347C, YOR356W, YOR374W, YPL034W, YPL248C, YPR026W, YPR086W, YPR189W

Supplemental Table S5A (continued)

S. Cerevisiae – S. paradoxus	Up	YBL076C, YBR008C, YBR199W, YDL160C, YDR107C, YDR158W, YDR273W, YDR321W, YDR378C, YDR441C, YDR533C, YEL009C, YEL027W, YER019C-A, YER027C, YER063W, YFL037W, YGL054C, YGL101W, YGL202W, YGL238W, YGR041W, YGR122W, YGR153W, YHR049W, YIL039W, YIL072W, YIL140W, YJL002C, YJL044C, YJL062W-A, YJL118W, YJR073C, YJR107W, YJR133W, YKL080W, YKL084W, YKL096W-A, YKL127W, YKL171W, YKL178C, YLR375W, YML112W, YOL036W, YPL037C, YPL066W, YPL127C
	Down	YBL029C-A, YBR120C, YBR147W, YBR281C, YCR063W, YCR071C, YCR083W, YDL078C, YDL181W, YDR078C, YDR408C, YDR438W, YEL024W, YEL041W, YER031C, YER048W-A, YGL136C, YGL191W, YGR006W, YGR049W, YGR096W, YGR102C, YGR165W, YGR209C, YHR059W, YHR168W, YIL070C, YIL128W, YIL160C, YJL146W, YJL218W, YJR119C, YLR100W, YML087C, YMR256C, YNR003C, YOL096C, YOL104C, YOR127W
		Gene Ontology Enrichment<br>Mitochondrion: CBP6/YBR120C, RTC2/YBR147W, IMG2/YCR071C, TRX3/YCR083W, INH1/YDL181W, THI74/YDR438W, RIP1/YEL024W, YPT31/YER031C,ISD11/YER048W-A, MRM2/YGL136C, COX13/YGL191W, SCM4/YGR049W, TPC1/YGR096W, GTF1/YGR102C, MRPS35/YGR165W,FYV4/YHR059W, MTG2/YHR168W, MAM33/YIL070C, ERG27/YLR100W, COX7/YMR256C, RPC34/YNR003C, COQ3/YOL096C
S. mikatae	Up	YAL008W, YAL009W, YAL060W, YAR008W, YBL025W, YBL029C-A, YBL057C, YBL106C, YBR053C, YBR085C-A, YBR103W, YBR195C, YBR273C, YBR280C, YCL033C, YCL034W, YCL035C, YCL049C, YCL057C-A, YDL181W, YDR166C, YDR223W, YDR322C-A, YDR364C, YDR377W, YER064C, YER068W, YER137C, YFL017W-A, YFL042C, YFR047C, YFR049W, YGL040C, YGL096W, YGL160W, YGL161C, YGL162W, YGL229C, YGR168C, YIL003W, YIL132C, YIR015W, YIR029W, YJL006C, YJL046W, YJL126W, YJL137C, YJL160C, YJL178C, YJL208C, YJL218W, YJR099W, YKL072W, YKL085W, YKL107W, YKL119C, YKL149C, YKL216W, YKR062W, YKR075C, YKR076W, YKR077W, YKR083C, YLR100W, YLR242C, YLR245C, YLR438C-A, YML131W, YMR027W, YMR101C, YMR113W, YMR148W, YMR174C, YMR251W-A, YMR293C, YNL164C, YNR028W, YNR037C, YOL114C, YOR227W, YPL098C, YPL170W, YPR023C, YPR094W, YPR157W
	Down	YAL022C, YAL035W, YBL091C, YBR158W, YBR180W, YBR199W, YBR250W, YCR017C, YDL198C, YDR036C, YDR184C, YDR373W, YDR523C, YEL013W, YEL027W, YER073W, YER086W, YER157W, YGL027C, YGL114W, YGL254W, YGR240C, YGR249W, YHR187W, YIL079C, YIL117C, YIR028W, YJR051W, YJR088C, YKL008C, YKR011C, YKR081C, YLR032W, YLR240W, YLR300W, YLR414C, YMR018W, YMR069W, YMR232W, YMR262W, YMR266W, YNL026W, YNL090W, YNL213C, YNL217W, YNL237W, YOL015W, YOR026W, YOR166C, YOR212W, YOR276W, YOR317W, YPL014W, YPL207W, YPR008W, YPR058W, YPR118W, YPR139C
		Gene Ontology Enrichment<br>nucleobase-containing compound transmembrane transporter activity: FUN26/YAL022C, GGC1/YDL198C, DAL4/YIR028W

Supplemental Table S5A (Continued)

S. bayanus	Up	YBL055C, YBL060W, YBR011C, YBR018C, YBR019C, YBR022W, YBR141C, YBR182C, YBR210W, YBR251W, YBR254C, YBR281C, YDL004W, YDL017W, YDL078C, YDL119C, YDL121C, YDL144C, YDR021W, YDR036C, YDR079W, YDR115W, YDR357C, YDR438W, YDR449C, YDR501W, YER147C, YER175C, YFR037C, YGL095C, YGL124C, YGL191W, YGL254W, YGR038W, YGR102C, YGR244C, YGR263C, YGR266W, YHL014C, YHR009C, YHR062C, YHR120W, YHR127W, YHR128W, YHR147C, YHR185C, YIL008W, YIL019W, YIL093C, YIL096C, YIL125W, YIL127C, YIL156W, YJL096W, YJL116C, YJL156C, YJL185C, YKL050C, YKL091C, YKL137W, YKL183W, YKR006C, YKR050W, YKR060W, YKR084C, YLR051C, YLR364W, YML087C, YML105C, YMR018W, YMR250W, YMR294W, YNL086W, YNL237W, YNL259C, YNL315C, YNL320W, YNL328C, YOL022C, YOL093W, YOL137W, YOR111W, YOR179C, YOR354C, YPL077C, YPL086C, YPR020W, YPR047W, YPR127W, YPR140W, YPR166C, YPR182W
	Down	YAL042W, YAR019C, YBL093C, YBL102W, YBR004C, YBR036C, YBR195C, YBR201W, YBR246W, YBR249C, YBR273C, YCL010C, YCL033C, YCL043C, YCL049C, YCR061W, YCR065W, YCR076C, YDL045W-A, YDL073W, YDL110C, YDL124W, YDL231C, YDL238C, YDR072C, YDR086C, YDR151C, YDR158W, YDR273W, YDR284C, YDR320C-A, YDR400W, YDR410C, YDR444W, YDR454C, YDR477W, YDR533C, YDR538W, YDR539W, YEL001C, YEL037C, YEL064C, YER018C, YER019C-A, YER035W, YER107C, YER134C, YFL010C, YFL045C, YFR044C, YFR050C, YFR052W, YGL048C, YGL063W, YGL130W, YGL167C, YGL175C, YGL196W, YGL198W, YGL250W, YGR026W, YGR204W, YGR234W, YGR253C, YHL020C, YHR020W, YHR049W, YHR100C, YHR179W, YHR189W, YIL076W, YIL095W, YIL109C, YIL116W, YIR029W, YIR036C, YJL062W-A, YJL068C, YJL153C, YJL194W, YJR019C, YJR067C, YJR075W, YJR107W, YJR133W, YKL047W, YKL072W, YKL096W-A, YKL127W, YKL210W, YKL211C, YKR026C, YKR075C, YLR027C, YLR098C, YLR268W, YLR325C, YLR349W, YLR372W, YLR375W, YLR380W, YML016C, YMR010W, YMR113W, YMR152W, YMR166C, YMR252C, YMR276W, YMR315W, YNL003C, YNL092W, YNL094W, YNL192W, YNL290W, YNR035C, YOL060C, YOR085W, YOR110W, YOR152C, YOR213C, YPL057C, YPL066W, YPL127C, YPL157W, YPL231W, YPL243W, YPL264C, YPL270W, YPR019W, YPR094W, YPR147C, YPR157W, YPR181C, YPR183W

Supplemental Table S5B: Genes DE in Each Branch of the Phylogeny (p<0.01 and FC>10)

	**Up**	**Down**
S. cerevisiae	YGR044C, YGR234W	YCL025C, YMR095C
S. paradoxus	None	YLR327C
S. cerevisiae – S. paradoxus	None	YIL160C
S. mikatae	YJL160C, YMR027W	YIL117C
S. bayanus	None	YJR107W

Supplemental Table S5A: Genes with lineage specific differential expression at $p<0.01$. Gene Ontology enrichment represents GO categories with enrichment at $p<0.01$. The complete set of core genes was used as the background dataset for gene enrichment analysis. When multiple categories encompassed the same gene set only the most significant category was listed. Overall, 353 genes were found to have increased in expression in a lineage-specific line, while 358 were decreased in expression relative to the other species. S**5B:** Genes with lineage-specific differential expression showing fold changes of at least 10X relative to each of the other four species.

Supplemental Table S6: The number of genes remaining after each inclusion criteria for potentially gene duplication events

	S. paradoxus	***S. mikatae***	***S. bayanus***
**Genes with multiple hits in species relative to S. *cerevisiae***	411	749	676
**Genes with two annotations in species**	309	566	514
**All ORFS are complete for the gene in this species (None go into unsequenced regions)**	126	146	195
**Not Duplicated in any other species (0 or 1 annotation)**	69	111	136
**More than 60% of the S. *cerevisiae* ortholog is covered by each potential paralog**	7	10	24
**Top BLAST to Cer Proteins Hit for Every Species**	5	9	17
**Expressed in S. *cerevisiae* and Species**	4	6	14
**90% of reads unique to annotation**	4	5	14

Supplemental Table S7: Measured Genes and the Reasons for Gene Exclusion by Species

				**Reasons For Exclusion**					
	**Annotated Orthologs**	**Measured**		**Length Differs by More than 10% Versus S. *cerevisiae* Ortholog**		**More than 10% of the reads aligned Non-uniquely**		**Non-Unique Annotation**	
S. paradoxus	4909	4359	89%	452	82%	89	16%	186	34%
S. mikatae	4401	3557	81%	467	55%	96	11%	343	41%
S. bayanus	4557	3628	80%	482	52%	183	20%	388	42%

Annotated Orthologs are the total number of genes annotated with a single ortholog in *S. cerevisiae* in the annotations of (Kellis et al. 2003). Numbers are the count of annotated ORFs. Non-unique annotations are *S. cerevisiae* genes that are annotated with more than one copy in the non- *S. cerevisiae* species. This may include both genes with paralogs and genes that are divided into two ORFs in the non-*S. cerevisiae* genome. Percentages are the percentage of genes that were excluded. The sum of these percentages is larger than 1 because some genes were excluded for multiple reasons. Reasons these exclusion criteria were chosen are described in Methods. Cross-species comparisons in non- *S. cerevisiae* species are made on the intersection of this set of measured genes.

Supplemental Table S8: The number of genes replicating within observed confidence intervals in same day versus different day comparisons

	**Same Day**		**Different Days**	
	**1σ**	**2σ**	**1σ**	**2σ**
S. cerevisiae- - S. paradoxus	55%	89%	35%	73%
S. cerevisiae- S. mikatae	54%	90%	33%	71%
S. cerevisiae- S. bayanus	46%	80%	34%	72%
S. paradoxus- S. mikatae	51%	86%	41%	79%
S. paradoxus- S. bayanus	59%	92%	33%	67%
S. mikatae- S. bayanus	45%	80%	37%	74%

S. *cerevisiae* Rep 1 calls against S. *paradoxus* Rep1 reproduced in S. *cerevisiae* Rep 2 against S. *paradoxus* Rep2. Different day comparisons are how well Rep1 calls against Rep 2 of the other species reproduced in Rep2 versus Rep1 of the other species.

Citations

1. Kellis M, Patterson N, Endrizzi M, Birren B, Lander ES: **Sequencing and comparison of yeast species to identify genes and regulatory elements**. *Nature* 2003, **423**(6937):241-254.
2. Quinlan AR, Stewart DA, Stromberg MP, Marth GT: **Pyrobayes: an improved base caller for SNP discovery in pyrosequences**. *Nat Methods* 2008, **5**(2):179-181.
3. Furusawa C, Kaneko K: **Zipf's law in gene expression**. *Phys Rev Lett* 2003, **90**(8):088102.
4. Tirosh I, Weinberger A, Carmi M, Barkai N: **A genetic signature of interspecies variations in gene expression**. *Nat Genet* 2006, **38**(7):830-834.
5. MacIsaac KD, Wang T, Gordon DB, Gifford DK, Stormo GD, Fraenkel E: **An improved map of conserved regulatory sites for Saccharomyces cerevisiae**. *BMC Bioinformatics* 2006, **7**:113.
6. Wang Y, Liu CL, Storey JD, Tibshirani RJ, Herschlag D, Brown PO: **Precision and functional specificity in mRNA decay**. *Proc Natl Acad Sci U S A* 2002, **99**(9):5860-5865.
7. Altschul SF, Gish W, Miller W, Myers EW, Lipman DJ: **Basic local alignment search tool**. *J Mol Biol* 1990, **215**(3):403-410.
8. Robinson MD, Oshlack A: **A scaling normalization method for differential expression analysis of RNA-seq data**. *Genome Biol* 2010, **11**(3):R25.
9. Bevington PR: **Data reduction and error analysis for the physical sciences**. New York,: McGraw-Hill; 1969.
10. Dallas PB, Gottardo NG, Firth MJ, Beesley AH, Hoffmann K, Terry PA, Freitas JR, Boag JM, Cummings AJ, Kees UR: **Gene expression levels assessed by oligonucleotide microarray analysis and quantitative real-time RT-PCR -- how well do they correlate?** *BMC Genomics* 2005, **6**(1):59.
